# Stress Corrosion Cracking: Mechanisms, Materials Challenges, and Engineering Solutions

**DOI:** 10.3390/ma19050898

**Published:** 2026-02-27

**Authors:** Lincoln Pinoski, Subin Antony Jose, Pradeep L. Menezes

**Affiliations:** Department of Mechanical Engineering, University of Nevada-Reno, Reno, NV 89557, USA; lpinoski@unr.edu (L.P.); subinj@unr.edu (S.A.J.)

**Keywords:** stress corrosion cracking, environmental degradation, mechanical integrity, electrochemical characterization, high entropy alloys

## Abstract

Stress corrosion cracking (SCC) is a critical failure mechanism that arises from the synergistic interaction between tensile stress and corrosive environments, leading to sudden and often catastrophic failures in structural components across various industries, including aerospace, nuclear energy, oil and gas, and marine engineering. This review synthesizes current understanding of SCC mechanisms, including film rupture and anodic dissolution, hydrogen embrittlement, and adsorption-induced cleavage, and evaluates material susceptibility across steels, aluminum alloys, nickel-based alloys, titanium, and emerging high-entropy alloys. Environmental factors such as aqueous chemistry, temperature, pressure, pH, and dissolved gases are examined for their roles in SCC initiation and propagation. Advanced testing methodologies, including slow strain rate testing, bent-beam configurations, electrochemical monitoring, and high-resolution microscopy, are discussed for characterizing SCC behavior. Engineering mitigation strategies are presented, encompassing material selection, stress reduction, surface treatments, and environmental control. Case studies illustrate real-world SCC failures and inform best practices. Emerging trends highlight the potential of machine learning for predictive maintenance and the development of SCC-resistant materials through additive manufacturing and microstructural engineering. This comprehensive review provides mechanical engineers with actionable insights for designing, maintaining, and safeguarding components against SCC in demanding service environments.

## 1. Introduction

### 1.1. Definition and Importance of SCC

Stress corrosion cracking (SCC) is the phenomenon by which tensile stresses in a material interact with a corrosive environment to initiate cracks, leading to material failure. Crucially, both factors, stress and a corrosive environment, must be present. Neither general corrosion nor static stress alone can fully predict SCC susceptibility. SCC failures often occur suddenly and without obvious warning, originating from areas where general corrosion may appear minimal [1]. This makes SCC particularly insidious in structural applications, since bulk corrosion rate measures do not reliably indicate the onset of crack initiation or rate of propagation [2].

### 1.2. Historical Perspective and Engineering Failures

**Figure 1:**
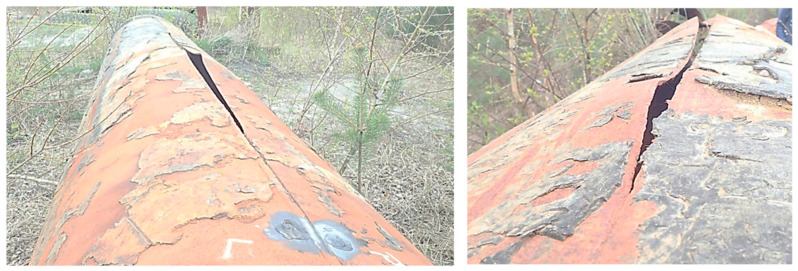
Failure of a water pipeline by SCC (axial cracks along the pipe length). Reproduced from [6], Materials, MDPI 2024.

SCC has led to major structural failures across several industries. In aerospace, high-strength aluminum alloys (e.g., 2024-T3, 7075-T6) used in airframes and fuselage frames have suffered SCC under cyclic pressurization and residual assembly stresses, prompting costly maintenance changes and even fleet groundings [3,4]. In the nuclear industry, coolant piping and joints between dissimilar metals have a high susceptibility to SCC due to high pressures and temperatures, galvanic coupling, and the presence of corrosive species in water. Also, pressurized water reactors have an elevated risk of hydrogen-assisted cracking due to the extreme pressures in the loop increasing rates of hydrogen entrapment, which requires careful control of water chemistry to limit the available hydrogen [5]. Oil and gas pipelines buried in moist soil or exposed to sulfide-rich fluids have experienced sudden ruptures from SCC (often under near-neutral pH conditions or in sour (H_2_S environments). Figure 1 shows the failure of a water pipeline caused by SCC, which occurred along the pipe length. These historical cases demonstrate that even when general corrosion is negligible or invisible, specific combinations of material vulnerability and environment can initiate cracks at stress levels far below the expected design limits, leading to catastrophic failures or the premature retirement of critical assets.

### 1.3. Relevance to Mechanical and Structural Components

Common structural alloys used in corrosive environments include austenitic stainless steels, high-strength low-alloy pipeline steels, 6xxx-series aluminum, and α-phase titanium. Each of these materials can be driven to its limits by service conditions, so durability and SCC resistance are vital considerations in design and maintenance. Components are often coated or cathodically protected to prevent general corrosion and extend service life. However, SCC can still occur due to local breakdown of those protections or unfavorable microstructural features. Because SCC failures impact safety and incur high economic costs (due to downtime and repairs, or injury in the worst cases), research into mitigating SCC and understanding its mechanisms remains a high-value area for mechanical engineers. High-profile failures in aircraft, bridges, and nuclear plants underscore that even advanced materials can suffer SCC if stress and environment align unfavorably [1,6,7].

## 2. Fundamentals of Stress Corrosion Cracking

### 2.1. Role of Stress and Corrosive Environments

SCC requires the simultaneous presence of tensile stress in the material and a corrosive environment. Tensile stresses may come from external loads or develop from manufacturing processes (e.g., welding, cold-forming) [8,9]. For example, in a bridge support beam, the underside experiences constant tensile stress from the structure’s weight, creating a predisposition for crack formation if the environmental conditions permit. These stresses concentrate at material discontinuities or “weak links” such as notches, grain boundaries, or cold-worked regions. The corrosive environment, meanwhile, chemically weakens the material locally. Aggressive species like chlorides, sulfides, or hydroxides attack protective films and the metal substrate, initiating pits or other defects that act as crack nucleation sites [10]. Importantly, the combined effect is synergistic, with the environment creating micro-scale flaws (e.g., pits, dissolved grain-boundary regions), and the tensile stress drives those flawed areas to evolve into cracks. As corrosion continues at the crack tip (for instance, by breaking down any repassivating film or generating hydrogen locally), the crack can progressively propagate, even under loads well below the material’s normal yield strength. Ultimately, the component can fail suddenly once the crack reaches a critical size, often with little visible corrosion elsewhere to warn of the danger.

### 2.2. Electrochemical and Mechanical Aspects

The corrosion aspect of SCC typically involves localized electrochemical reactions. For example, in many alloys, a protective oxide film provides resistance to general corrosion. SCC often begins with the anodic dissolution of this passive film at a highly stressed point, leading to the formation of a tiny pit or etched area. In the presence of chloride ions, this “film rupture” can happen repeatedly, where a film breaks under stress, and the underlying metal dissolves anodically until the film reforms, then the cycle repeats, effectively etching a crack path into the material (this is known as the slip-dissolution or film rupture mechanism) [11]. Mechanically, the material’s properties (strength, ductility, microstructure) determine how easily cracks will initiate [12]. Ductile materials can tolerate some crack tip plasticity without fracturing, possibly slowing crack growth, whereas brittle or high-strength materials may fracture more readily once a crack exists. Defects from processing (weld inclusions, heat-affected zones, cold-worked regions) can serve as initiation sites or aid crack propagation by creating stress concentrations. SCC is a complex interplay, with the corrosion process continuously feeding the crack (by dissolving metal or producing embrittling hydrogen) and the mechanical stress continuously driving the crack forward.

### 2.3. Distinction Between SCC, Corrosion Fatigue, and Hydrogen Embrittlement

It is important to separate SCC from related phenomena like corrosion fatigue (CF) and hydrogen embrittlement (HE). CF involves cyclic stresses in a corrosive environment; cracking occurs from the synergy of repeated loading and corrosion, even if each acting alone may not cause failure [13]. HE occurs when atomic hydrogen infiltrates a metal (often under static load or cathodic polarization), leading to brittle fracture without the need for an externally applied cyclic stress [14]. In contrast, classical SCC requires a steady tensile stress and a specific corrosive medium, often resulting in cracks that are either intergranular (along grain boundaries) or transgranular (through grains) with features of both corrosion and mechanical failure at the crack surfaces [6]. Table 1 contains a summary of the unique properties for each failure mode.

## 3. Mechanisms of SCC

### 3.1. Film Rupture and Anodic Dissolution Model

**Figure 2:**
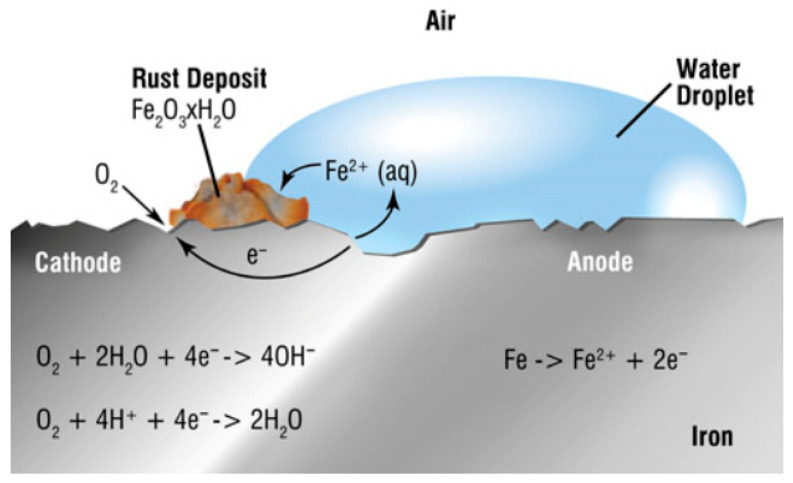
Anodic dissolution and film rupture mechanism causing SCC in iron. Reproduced from [17], Metals, MDPI 2019.

Many alloys rely on a thin, protective oxide film (passive film) for corrosion resistance. Under tensile stress, localized plastic deformation can rupture this film repeatedly. Each time the film cracks, the exposed metal beneath is anodically dissolved by the environment until the film reforms [15]. This cycle of slip and dissolution produces a fine, stepwise advancement of a crack. Over time, the crack deepens as metal is incrementally removed at the crack tip during each rupture–re-passivation event [16]. This mechanism is common in metals that depend on passivity, such as austenitic stainless steels, aluminum alloys, and nickel-based alloys in hot chloride solutions [1]. The cracks can be transgranular or intergranular. Figure 2 illustrates anodic dissolution leading to SCC on iron (transgranular cracking with corrosion product deposits). Significant corrosion at crack tips (etched or corroded appearance inside the crack) is evidence of dissolution-driven growth. Mitigation of this mode involves enhancing film stability (using Mo-containing stainless steel to resist chloride attack) and reducing stress or strain localization that cause film rupture.

### 3.2. Hydrogen Embrittlement Model

**Figure 3:**
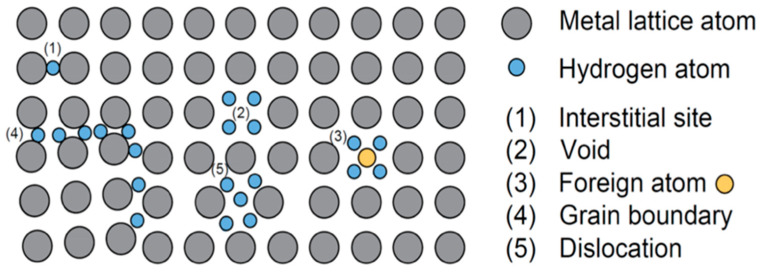
Schematic of hydrogen embrittlement processes. Reproduced from [19], Energies, MDPI 2025.

In some environments, particularly those that promote hydrogen generation (like cathodic protection or acidic conditions), metals can become brittle when hydrogen atoms diffuse into the microstructure. Hydrogen embrittlement (HE) can accompany SCC by two primary sub-mechanisms, hydrogen-enhanced decohesion (HED) and hydrogen-enhanced localized plasticity (HELP). In the HED view, hydrogen reduces the atomic bond strength at the crack tip, causing normally ductile metal to develop brittle fractures. In the HELP view, hydrogen concentrated at dislocations and crack tips enables dislocations to advance, leading to localized slip and micro-void formation that accelerates crack growth. The net effect of both is metal exhibiting brittle cracking (cleavage or intergranular fracture) even while the bulk material remains ductile. This mechanism is often involved in SCC of high-strength steels and titanium alloys in environments that provide hydrogen (like sour oil/gas service or seawater service under cathodic protection) [18]. For instance, pipeline steels can suffer sulfide stress cracking. H_2_S in the environment generates hydrogen on the steel surface, which then embrittles the steel and causes intergranular cracking under stress. Figure 3 shows the mechanisms of hydrogen embrittlement (grain-boundary decohesion due to hydrogen). Prevention focuses on minimizing hydrogen ingress (properly controlled cathodic protection potentials, use of inhibitors, baking to remove hydrogen) and using alloys or heat treatments that trap hydrogen in less harmful forms (forming innocuous hydrides or carbide traps).

### 3.3. Adsorption-Induced Cleavage Model

**Figure 4:**
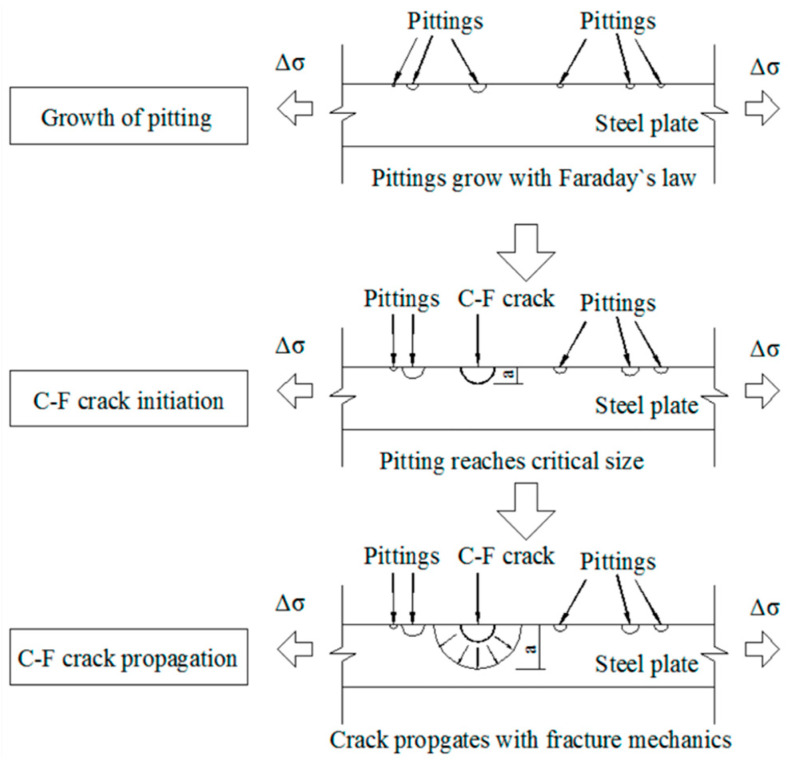
Characteristic stages of corrosion fatigue crack initiation and propagation. Reproduced from [21], Applied Sciences, MDPI 2019.

In this mechanism, certain aggressive species in the environment (commonly Cl^−^, but also OH^−^ and others) specifically adsorb into the crack tip or highly stressed regions of the metal lattice and lower the surface energy or bond strength. This adsorption-induced cleavage (AIC) embrittles the surface atomic layers without requiring significant hydrogen uptake. The result is a brittle fracture along cleavage planes. Essentially, the metal cracks because the adsorbed atoms (like chloride) make it easier for atomic bonds to snap under stress [20]. AIC can cause transgranular cracks that look like pure brittle fractures (shiny cleavage facets) even in otherwise ductile materials. Unlike hydrogen embrittlement, AIC does not require hydrogen penetration; it is a surface effect. This model is supported by observations that certain environments cause cracking in materials that do not easily absorb hydrogen. For example, stress corrosion cracking of mild steel in hot nitrate solutions is thought to proceed by an adsorption mechanism, as nitrates adsorb on iron and induce brittle cracking without hydride formation. Figure 4 outlines the stages of corrosion fatigue crack growth (which typically initiates at a pit and advances incrementally with each load cycle). Mitigating AIC-type SCC involves removing or substituting the aggressive ions in the environment (using inhibitors or different fluids) and reducing the tensile stress state. Even small concentrations of aggressive ions can dramatically increase the SCC risk. Stringent purity and monitoring of process fluids (limiting chlorides to only a few ppm in boiler feedwater) is a common strategy for prevention.

**Table 1:** Summary of SCC mechanisms: AIC, HE, FR/SD.

Mechanism	Primary Driving Agent	Crack Path Morphology	Mitigation Strategies	References
Adsorption-Induced Cleavage (AIC)	Specific adsorption of aggressive ions (e.g., Cl^−^, S^2−^, OH^−^) at highly stressed surface sites weakens metallic bonds and reduces surface energy, promoting brittle cleavage	Predominantly transgranular with cleavage-like facets (bright, mirror-like fracture surfaces) and minimal plastic deformation (a brittle appearance)	Remove or neutralize aggressive ions (use purified water or inhibitors), maintain controlled environment chemistry (pH buffers, de-aeration), reduce applied tensile stress (stress relief, design changes), and improve alloy grain-boundary cohesion (via microalloying or heat treatments)	[20,21]
Hydrogen Embrittlement (HE)	Absorption and diffusion of atomic hydrogen into the metal lattice; hydrogen accumulates at stress concentrators (dislocations, grain boundaries, inclusions), reducing cohesive strength or enhancing localized plasticity (via HEDE or HELP models)	Often intergranular in high-strength steels (due to hydrogen at grain boundaries), or transgranular quasi-cleavage with secondary microcracks. Hydride-forming alloys (Ti, Zr) show brittle hydride cracking along grain boundaries	Prevent hydrogen ingress (apply coatings or diffusion barriers, optimize cathodic protection to avoid over-charging with hydrogen), perform bake-out heat treatments to remove absorbed H, lower material strength/hardness if possible, use microstructures or alloying additions that trap hydrogen in benign forms (e.g., adding Ti or Mo to steel to form stable carbides that trap H)	[18,19]
Film Rupture/Slip–Dissolution (FR/SD)	Cyclic rupture of a passive oxide film at the crack tip due to localized plastic strain, exposing fresh metal that undergoes anodic dissolution before the film re-passivates. Repetition of film breakage and reformation drives crack advance	Typically transgranular, often showing striations or etched facets where the metal dissolved. Can sometimes become intergranular if grain-boundary precipitates or segregation cause preferential attack	Use alloys that maintain a stable passive film under stress (e.g., add Mo or N to stabilize stainless steel films), reduce surface tensile stress and strain localization (polish surfaces, avoid sharp notches), use inhibitors that promote rapid re-passivation (e.g., molybdate, nitrate), and avoid sensitization heat treatments that would weaken grain-boundary film integrity	[15,16,17]

## 4. Material Susceptibility to SCC

Different materials exhibit varying susceptibilities to SCC depending on their composition, microstructure, and strength. This section discusses key material classes and how they respond to SCC, highlighting which alloys are prone to SCC and why, as well as any advanced alloys designed for improved SCC resistance.

### 4.1. Steels

Austenitic stainless steels (304, 316L) are somewhat susceptible to chloride-induced SCC, especially at elevated temperatures (>50–60 °C) or if sensitized (grain boundaries depleted of chromium). For instance, 304SS has been observed with crack growth rates between 10^−7^ and 10^−8^ m/s in 40% artificial seawater at elevated temperatures of more than 50 °C. These conditions are a realistic representation of brine exposure from salt deposition on structures in near-coastal areas [22]. The 316L used in nuclear reactor cooling systems can crack in hot chloride or boric acid environments due to film breakdown and localized corrosion at stress concentrators. That said, stainless steels retain excellent toughness and weldability, with their SCC vulnerability mainly in specific conditions (chlorides, high temperature) [23]. By contrast, duplex stainless steels (e.g., 2205, 2507), which have a mixed ferrite and austenite microstructure, generally have better pitting and SCC resistance in chloride environments up to 100–150 °C [24]. They can, however, still suffer SCC in hot chloride conditions or if exposed to conditions causing hydrogen uptake (duplex steels under cathodic protection in sour service can crack via hydrogen embrittlement) [1]. pH plays an important role in the passive film stability and threshold stress intensity factor of stainless steels by controlling hydrogen evolution. In acidic environments (low pH), hydrogen evolution is enhanced, increasing hydrogen ingress. As more hydrogen is absorbed the threshold stress intensity factor is lowered, which greatly increases crack growth rates. In acidic conditions (high pH), carbon steels experience caustic cracking more readily due to passive film instability and increases in local dissolution [18,25]. Proper phase balance and nitrogen content in duplex steels help maximize SCC resistance. Martensitic stainless steels (like 410 or 420) are very strong but less corrosion-resistant; they typically are used where hardness is needed, and SCC risk is managed by controlling the environment (they are more prone to general corrosion and thus to SCC if unprotected).

High-strength low-alloy pipeline steels (HSLA steels like X52, X70) can undergo SCC in near-neutral pH groundwater environments. These SCC cases usually occur at coating defects where the steel is exposed to a wet soil environment under residual and hoop stresses. Microstructural cleanliness (low inclusions) and appropriate heat treatments improve their resistance. HSLAs can also suffer caustic embrittlement (a form of SCC) in concentrated hydroxide solutions at high temperatures (boilers, heat exchangers).

### 4.2. Aluminum Alloys

Aluminum alloys vary widely in SCC behavior. The 2xxx series (Al-Cu-Mg, 2024) and 7xxx series (Al-Zn-Mg-Cu, 7075) are high-strength aerospace alloys that are quite susceptible to SCC under certain conditions. In 2xxx alloys, grain-boundary Cu-rich precipitates can undergo anodic dissolution in moist environments, leading to intergranular SCC under sustained stresses [26,27]. The 7xxx alloys in peak-aged tempers (like 7075-T6 or 7050-T7451) are particularly prone to SCC in the presence of water or salt, especially if they contain coarse grain-boundary precipitates or precipitate-free zones that promote localized attack [28]. Measures like over-aging (to more ductile tempers) or using newer alloys (like 7050 or 7475 with Zn, Mg, Cu, plus Zr/Cr to refine grain-boundary precipitates) can improve the SCC resistance of 7xxx alloys. The 5xxx series (Al-Mg, 5083) typically has good SCC resistance unless it is sensitized by prolonged exposure to moderate temperatures (70–150 °C). In sensitized 5xxx, a Mg-rich β phase (Al_3_Mg_2_) can form along grain boundaries and is anodic, leading to intergranular corrosion and SCC (for instance, in welded 5083 marine alloys used in ship hulls) [29]. The 6xxx series (Al-Mg-Si, 6061) generally has low to moderate SCC susceptibility, is medium-strength, and forms Mg_2_Si precipitates that do not severely weaken grain boundaries; thus, 6xxx alloys are commonly used for extrusions and show decent SCC resistance unless aggressively attacked by chlorides with sustained stress [30,31]. The 6000 series aluminum has a higher threshold stress intensity factor compared to 7000 series alloys, leading to slower crack growth in comparable conditions. Modern aluminum–lithium (Al-Li) alloys (like 2195) offer high strength-to-weight and improved SCC resistance compared to 2xxx/7xxx alloys. The addition of Li in these alloys changes the precipitate structure (forming strengthening phases that do not promote continuous grain-boundary networks) and increases the material’s inherent resistance to fatigue and SCC. Al-Li alloys have been successfully used in aerospace applications (the Space Shuttle’s 2195 Al-Li external tank) with great performance [32]. pH and temperature play an important role in SCC of aluminum alloys, affecting passive film stability and local corrosion kinetics. The Al_2_O_3_ film is unstable or soluble in alkaline or acidic solutions, causing pit formation, which can lead to cracks. Increased temperature accelerates the breakdown of the passive film, leading to reduced time to failure and increased crack growth rate.

Across the aluminum alloys, grain-boundary precipitate spacing plays an important role in determining SCC susceptibility in chloride environments [33]. GBP spacing is strongly correlated with temper and yield strength in 2xxx, 6xxx, and 7xxx series alloys, which can be precipitation hardened. Generally, a narrow or continuous precipitate spacing is more susceptible to SCC; these precipitates provide pathways for anodic dissolution between grains, accelerating crack propagation. Narrow precipitate spacing usually increases yield strength by limiting dislocations within the microstructure, meaning there can be a correlation, especially within the same series of alloys, between higher yield strength and increased SCC susceptibility [34]. This trend is explored further in Table 2, showing crack propagation rates reported for two aluminum alloys, 6005A and 2024-T3, in comparable environments of 3.5% NaCl. The 2024-T3 is reported to have a slightly higher crack propagation rate than the 6005A alloy and a higher yield strength. When hardened, 2024 aluminum forms primarily copper-based S-phase Al_2_CuMg precipitates, which provide increased strength but are very susceptible to corrosion in chloride environments [35]. The 6005 alloy is comprised primarily of aluminum (~98%) with silicon (~0.8%), magnesium (~0.7%), and manganese (~0.4%) [36]. This results in 6005 having a lower yield strength compared to 2024, but with fewer precipitates that are susceptible to corrosion, increasing SCC resistance.

### 4.3. Nickel-Based Alloys

Nickel-based alloys (Inconel, Monel, Hastelloy) are generally designed for excellent corrosion and SCC resistance in harsh environments. Inconel 600/625/718 (Ni-Cr or Ni-Cr-Fe) are used in nuclear reactors, chemical plants, and turbines and have high resistance to chloride SCC, though they can suffer caustic cracking or primary water SCC (PWSCC) in nuclear primary water over long periods. Alloy 600, for instance, exhibited SCC in nuclear steam generator tubing (high-temperature, high-purity water with oxygen), leading to a switch to Alloy 690 (higher Cr), which is far more SCC-resistant in those conditions. Hastelloy C-276 (Ni-Cr-Mo) and similar alloys have among the highest resistances to SCC in hot acidic or sour environments; the Mo addition in particular helps prevent localized attack even in boiling chloride or sulfuric acid solutions. Monel 400 (Ni-Cu) is highly resistant to seawater SCC (used in marine engineering), but in strongly oxidizing acids, it can exhibit grain-boundary attack and cracks. Generally, Ni alloys are chosen when standard steels or bronzes would crack; oil and gas sour service uses Incoloy 825 or Inconel 718 for tubulars to avoid sulfide stress cracking [37,38]. They are not immune to SCC, but their threshold for SCC is very high (requiring extremely aggressive conditions). As a trade-off, Ni alloys are expensive and can be challenging to fabricate.

### 4.4. Titanium and Advanced Alloys

Titanium alloys are notable for combining high strength, low density, and excellent corrosion resistance. Commercially pure titanium (grades 1–4) and α-type Ti alloys (like Ti-5Al-2.5Sn) have very high SCC resistance in most environments, including chlorides, due to the strong, self-healing TiO_2_ passive film [39,40]. Unalloyed Ti is used for seawater cooling systems and generally does not suffer SCC in chloride media (unlike steels or Al). α + β titanium alloys (like Ti-6Al-4V) also have good SCC resistance in neutral and mildly acidic environments, but in the presence of certain species like methanol or under cathodic protection (hydrogen charging), even Ti-6Al-4V can form brittle hydrides leading to crack initiation [41]. For example, Ti alloys in deep sour wells or under improper cathodic protection may experience hydrogen embrittlement cracking. Beta-titanium alloys are used less in corrosive service because they often contain elements that reduce corrosion resistance. Generally, titanium’s primary SCC concern is hydrogen embrittlement (hydride formation) rather than film rupture or dissolution; keeping Ti out of conditions that cause hydrogen uptake (like over-negative potentials) prevents SCC in Ti components. Compared to more common structural materials like steels and aluminum, titanium SCC is governed by hydrogen-assisted mechanisms rather than anodic dissolution, leading to very low crack growth in neutral chloride environments. Far from neutral pH and elevated temperatures can increase hydrogen evolution and lead to cracking due to a lowered threshold stress intensity factor [39,42].

An emerging class of materials, high-entropy alloys (HEAs), and other complex concentrated alloys contain multiple principal elements (often five or more) and are being studied for their potentially superior corrosion and SCC resistance. Early research suggests that some HEAs form exceptionally strong passive films and may have high resistance to both localized corrosion and hydrogen embrittlement, though most are still in the prototype stages for structural applications. For example, certain HEAs have shown no SCC in boiling 3.5% NaCl, where conventional stainless steels would crack, thanks to complex oxides enriching their surfaces. Development continues, focusing on optimizing phase stability and eliminating minor phases that could act as initiation sites. Table 3 below compares several material classes in terms of SCC susceptibility and influencing factors.

### 4.5. Corrosion Potentials

Corrosion potential can be linked to SCC, but cannot directly predict failure times or SCC susceptibility. However, it is important to consider the corrosion potential of a material selected for an environment. A common environment for both testing and in real-world application is a 3.5% NaCl solution, usually seawater, at room temperature, closely resembling real-world conditions [30]. This provides a useful baseline for accessing conditions leading to anodic dissolution and film rupture mechanisms that may contribute to crack initiation under tensile stress. Table 4 gives an overview of the typical corrosion potential of materials listed in the previous sections, including common structural steels, aluminum alloys, titanium, and Ni alloys. Corrosion potential is dependent on many factors, including electrolyte, temperature, pH, surface finish, and chloride concentration. However, the corrosion potential in a 3.5% NaCl environment serves as a useful baseline for comparison between materials [47]. Corrosion potential is represented as E_corr_ in volts; a more negative potential has a high anodic dissolution tendency, leading to film rupture and a high risk of cracks under tensile stresses.

## 5. Influence of Environmental Factors

### 5.1. Aqueous Environments

**Figure 5:**
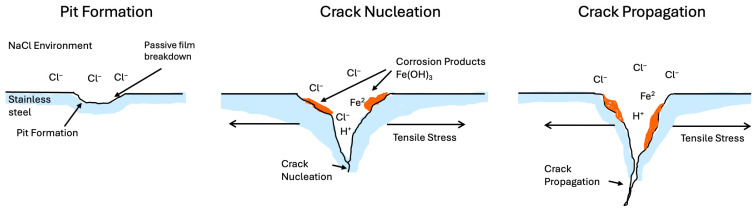
Schematic of chloride-induced SCC mechanism of stainless steel (passive film breakdown and pit formation, leading to crack and failure).

Water-based environments (especially those containing chlorides) are notorious for causing SCC in susceptible alloys. Chloride ions (Cl^−^) are the most common triggers, as they break down passive films on metals like stainless steel or aluminum, leading to pits that serve as crack nucleation sites [54]. For example, high-temperature chloride-bearing water can induce SCC in 300-series stainless steels even if general corrosion is low. Seawater is a prime example of an aggressive medium containing chlorides that facilitate SCC in steels and aluminum alloys. Figure 5 shows the evolution of SCC on P92 steel in a chloride-rich (seawater) environment. Additionally, seawater can harbor biofouling and microbiologically influenced corrosion (MIC) [55].Organisms deposit sulfides and create differential aeration cells, altering the local chemistry (producing H_2_S or local acidity) and thereby accelerating pitting and crack initiation [56]. (For instance, stainless steel subsea components may pit under biofilms, and those pits become SCC crack starters.) In practice, marine structures often use cathodic protection (CP) or sacrificial zinc anodes to prevent general corrosion. However, one side effect is that overly aggressive CP can generate hydrogen on a steel surface, potentially leading to hydrogen embrittlement SCC in high-strength steels striking a balance in CP potential, which is critical. Another aqueous environment is caustic solutions (high-concentration hydroxide, such as concentrated NaOH). Carbon steels and low-alloy steels can suffer “caustic embrittlement” or caustic SCC in boiler systems when concentrated hydroxide plus tensile stress causes intergranular cracking [57]. Preventive measures include controlling water chemistry (limiting hydroxide concentration and impurities) and stress-relieving welds in boiler tubing. In summary, wet environments, especially those high in chlorides or extremely acidic and basic, tend to promote the breakdown of protective films and the generation of local corrosion sites that lead to SCC. Flow conditions also matter, as low-flow stagnant zones encourage crevice corrosion that can initiate SCC, whereas high flow may erode protective films and promote SCC (by continuously removing passivating films) [6].

### 5.2. Influence of Temperature and Pressure

Elevated temperature generally accelerates SCC processes. Higher temperatures increase the diffusion rates of corrosive species and the kinetics of electrochemical reactions, which can lead to faster passive film breakdown and more rapid crack growth [58]. For example, a stainless steel that is immune to SCC at room temperature may start cracking in hot water above 60 °C because the protective film cannot reform quickly enough after ruptures, or because certain deleterious phases become active. Pressure itself (if static) mainly contributes via stress, but high pressure can also increase the solubility of gases like oxygen or hydrogen in water. In a pressurized system (like a pressurized water reactor or deep-sea pipeline), more dissolved O_2_ can shift corrosion potentials more noble, driving anodic dissolution at cracks. Similarly, high pressure H_2_S/CO_2_ in oil wells increases hydrogen uptake in steels, exacerbating sulfide stress cracking. A practical example is that deep oil wells and geothermal wells, which operate at high temperatures and pressures, see significantly higher SCC and general corrosion rates, diffusion of hydrogen into steel is enhanced, and passive films (like iron carbonate scales) become less stable [59]. The mechanical effects of high temperature include possible creep or stress relaxation, which can either relieve some stress or, conversely, create stress concentration if differential expansion occurs in assemblies. In a welded structure with constrained geometry, heating can cause thermal stresses that contribute to SCC. For example, differential expansion between a stainless steel pipe and a steel flange at high temperature can put extra stress on bolts, leading to SCC in those bolts if exposed to steam [60]. Under combined high temperature and pressure, the “aggressiveness” of the environment often increases nonlinearly. For example, supercritical water at 400 °C not only speeds up corrosion reactions but also can change the corrosion product chemistry, leading to a loss of protection and rapid crack propagation. Table 5 summarizes the primary environmental factors that can accelerate SCC and crack propagation in most materials.

Laboratory tests back these observations. Galakhova et al. [59] found that raising temperature and pressure dramatically reduced the time-to-crack initiation for OCTG (oil country tubular goods) materials in simulated downhole conditions, confirming that SCC activation energy is readily overcome at high temperatures. In summary, designers must treat stated “SCC-resistant” performance as temperature-dependent, where materials that perform well at ambient conditions may require derating or additional protection at higher operating temperatures and pressures.

### 5.3. Role of pH, Dissolved Oxygen, and Specific Ions

Solution pH and dissolved gases strongly influence SCC by altering corrosion mechanisms. Low pH (acidic conditions) tends to increase hydrogen evolution (cathodic reaction), thereby promoting hydrogen embrittlement contributions to SCC. For example, pipeline steels in near-neutral pH (≈6) groundwater suffer SCC via a mechanism involving hydrogen (from bicarbonate/carbonate reactions), whereas in high-pH (>10) concentrated carbonate solutions, a different intergranular crack mechanism occurs. High pH (strongly alkaline) can cause certain alloys (like carbon steel) to undergo caustic cracking. This occurs when the steel’s protective magnetite film dissolves in hydroxide, and atomic hydrogen is generated, leading to intergranular cracks. This typically occurs in riveted steam boilers, where concentrated NaOH formed at stay-bolt pockets causes failures. Dissolved oxygen (DO) serves as a cathodic reactant, as elevated DO typically raises the corrosion potential of a metal, which can push stainless steels or Ni alloys into the trans passive regime where rapid film breakdown and SCC can occur. In practical terms, oxygenated high-temperature water in power plants accelerated SCC of Alloy 600 tubing, whereas adding hydrogen (to scavenge oxygen) and thus de-aerating the water greatly mitigated that form of cracking. This is why boiler feedwater and nuclear reactor coolants are often de-oxygenated (via chemical scavengers or vacuum de-aerators); reducing DO content slows down pitting and SCC in stainless steels and nickel alloys. Specific ions aside from chloride can also contribute to degradation. Nitrates (NO_3_^−^) can cause SCC in carbon steels at high temperatures by an adsorption–cleavage mechanism; hydroxides (OH^−^) in caustic cracking of steels, sulfide (S^2−^) and bisulfide (HS^−^) facilitate hydrogen entry and cause sulfide stress cracking in steels (a form of SCC) [61]. Even minute levels of impurities can be enough, as trace chloride or sulfate in a high-purity system can accumulate under deposits or in crevices and trigger SCC. Even tiny amounts of impurities can be enough, as trace chloride or sulfate in a high-purity system can build up in deposits or crevices and cause SCC. Therefore, among other prevention methods, managing water chemistry (pH adjusters, O_2_ scavengers, ion purification) is one of the most effective ways to prevent SCC.

**Table 5:** Environmental factors affecting SCC and examples of component failure.

Environmental Factor	Mechanism/Influence	Effect on SCC	Example Environments and Materials	References
Chloride Ions (Cl^−^)	Break down passive films and foster pitting; chloride anions adsorb on metal and promote localized anodic dissolution.	Accelerate crack initiation and propagation by creating pit nuclei and maintaining active crack tip dissolution.	Seawater (SCC of 304 SS, 7000-series Al); chloride process streams attacking stainless reactor components.	[54,55,56]
High Temperature	Increases corrosion kinetics and diffusion rates; can destabilize passive films and cause thermal stress gradients.	Greatly speeds up SCC growth and can lower the threshold stress for SCC by enhancing chemical attack at crack tips.	Nuclear reactor water (~300 °C, SCC in Alloy 600); steam boilers (caustic + high T causing cracking in carbon steel).	[58,59,60,61]
High Pressure	Raises gas solubility (O_2_, H_2_) in liquids and can increase true stress intensity on flaws (if pressure-containing).	Can lead to more H_2_ absorption (thus more HE) and higher crack-driving force. Often coupled with high T effects.	Deep-sea pipeline (high external pressure, promoting H_2_ uptake under CP); PWR reactor primary loop (15 MPa, more dissolved H_2_/O_2_ affecting Alloy 690).	[60,61]
Low pH (Acidic)	Promotes hydrogen evolution (cathodic reaction) and dissolves protective oxides.	Increases risk of hydrogen embrittlement SCC and general acid attack that pre-pits the material.	Sour petroleum fluids (pH ~3–5, steel tubing SCC); acid pickling residues on high-strength steel.	[54,55,56,57]
High pH (Caustic)	Causes certain alloys (e.g., carbon steel) to form soluble compounds; can generate atomic H at metal surface.	Leads to caustic cracking (intergranular SCC in steels) if tensile stresses are present.	Boiler caustic environments (NaOH in riveted steam drums); alkali process vessels.	[56,57]
Dissolved Oxygen	Increases cathodic reduction rates (oxygen reduction) and raises corrosion potential.	Shifts conditions toward active cracking for alloys like stainless steel by supporting continuous film rupture corrosion cycles.	Aerated vs. de-aerated water in power plants (Alloy 600 SCC occurred in oxygenated water, mitigated when DO removed).	[6]
Sulfide/H_2_S	Poison catalysts for hydrogen recombination, leading to more hydrogen entering the metal; also forms metal sulfides (often less protective).	Causes sulfide stress cracking in steels (a type of hydrogen-assisted SCC) even at mild pH. Embrittles grain boundaries.	Oil & gas sour environments (pipeline steels cracking in H_2_S-containing brines).	[61]
Flow Conditions	Low flow leads to stagnant crevices; high flow causes erosion of passive film and constant supply of reactants.	Low flow: Crevice corrosion initiates cracks. High flow: Strip protective films, possibly higher corrosion currents at crack tip.	Crevices under deposits in seawater heat exchangers (low-flow SCC); high-velocity steam or slurry causing film damage in piping.	[6]

After exposure to aggressive environments, the experiments show that higher temperature and pressure dramatically shorten the time to SCC [62,63]. For example, Lopez-Dominguez et al. [63] observed that corrosion currents for a T91 steel in molten salt increased by an order of magnitude as the temperature rose, leading to rapid crack formation. Similarly, constant-extension tests on alloy 182 weld metal found that SCC initiation in high-purity water has a definitive activation temperature, where below it shows no cracking and above it SCC cracks form quickly. These findings underscore the importance of testing materials under actual service conditions (temperature, pressure, chemistry) rather than relying on ambient tests, as SCC behavior can change drastically with the environment [59]. In practical engineering, environmental control is a primary defense. Using inhibitors or water chemistry control (adding oxygen scavengers like hydrazine in boiler water to remove dissolved O_2_, or maintaining a slightly alkaline pH in secondary loops) reduces SCC propensity. Alloy/coating selection is often dictated by the expected environment, e.g., using titanium or super duplex stainless steels for seawater service, or applying thermal spray coatings on steel in molten salt reactors to protect against extreme corrosives [64].

## 6. Testing and Characterization of SCC

Engineers and researchers employ a variety of methods to test materials for SCC susceptibility and to characterize cracks. These include specialized mechanical tests in corrosive environments, post-test microscopic analysis, and in situ monitoring techniques to detect cracking as it occurs.

### 6.1. Experimental Methods

Constant Load Testing: A sample (often a tensile specimen or a C-shaped ring) is loaded in tension at a constant stress level and exposed to a corrosive medium for an extended time. The time to failure (if it fails) or the threshold stress below which it does not fail is recorded. Constant load SCC tests can run for thousands of hours. They directly simulate long-term service conditions but are time-consuming. For example, a steel might be loaded to 50% of its yield strength in a hot chloride solution; if SCC is going to occur, the specimen may crack after 1000 h. If it survives a designated test period (e.g., 5000 h) without cracking, that stress level might be deemed safe. One challenge is maintaining a truly constant load despite creep or relaxation; lever arms or spring-loaded fixtures are often used [65].

Slow Strain Rate Testing (SSRT): In an SSRT, a tensile specimen is pulled at an extremely low strain rate (such as 1 × 10^−6^ to 1 × 10^−7^ newtons per second) to failure in a corrosive environment. The idea is that if the material is SCC-susceptible, it will exhibit a markedly lower elongation to failure and reduced time-to-fracture compared to the same test in an inert environment (or compared to a more SCC-resistant alloy). SSRT is relatively quick (taking hours or days, rather than weeks) and sensitive to SCC; a material that undergoes SCC will often fail at a stress well below its normal fracture stress and with a brittle appearance. Lower strain rates generally promote SCC because they allow more time for corrosion processes during straining. For example, an SSRT on sensitized 5083 aluminum alloy in saltwater showed a sharp drop in ductility and clear intergranular fracture morphology, indicating SCC, whereas an unsensitized sample maintained good ductility [66]. SSRTs are convenient for comparing materials or environments, though they may not perfectly replicate the service stress state (since actual service is usually constant load or cyclic, not a continuous slow ramp).

**Figure 6:**
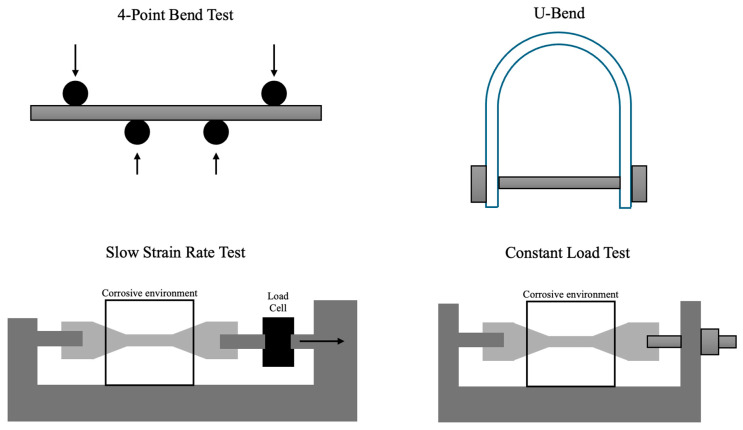
Schematics of common SCC testing strategies: 4-point bend, U-bend, SSRT, and constant load. Arrows indicate applied stresses.

Bent-Beam Tests (U-bend, C-ring): In these classic tests, a specimen is bent into a curve, creating a region of high tensile stress, and then exposed to the environment [67]. For instance, a U-bend test takes a strip of metal bent 180° and held in that bent configuration (as per ASTM G30). Such specimens have very high stress on the outer surface of the bend, often above yield, which accelerates SCC if the material is susceptible. Figure 6 shows diagrams of common U-bend and C-ring configurations. Multiple U-bend coupons can be placed in a test solution to see if cracks appear over time. U-bend and C-ring tests are easy and quick screening tools (often used in alloy qualification). The presence of cracks (viewed after the test under a microscope) indicates SCC susceptibility qualitatively. However, the stress distribution is non-uniform (highest at the bend apex), so these tests do not provide quantitative data like threshold stress intensity [68]. They are essentially pass/fail. For example, a U-bend of 304 stainless might crack in 42% boiling MgCl_2_ within 24 h (a standard test environment for screening stainless SCC), whereas a U-bend of alloy 690 (high Ni) would remain uncracked—indicating the much higher SCC resistance of the Ni alloy.

Four-Point Bend Test: A beam sample is supported and loaded in a four-point bending jig while immersed in a corrosive environment. This creates a constant bending moment (and thus a relatively uniform tensile stress over a large central region of the sample). Four-point bending SCC tests allow more controlled stress levels than U-bends. Researchers can monitor crack growth lengthwise along the beam. For instance, a recent study used a four-point bent plate of pipeline steel with an attached probe to monitor crack growth in situ and could quantify the crack growth rate under known stress intensity conditions. Four-point tests provide quantitative crack growth data and can be run with potential control (for electrochemical monitoring) but require more complex fixtures and sometimes take longer than U-bend tests for cracks to initiate [15].

Corrosion Fatigue Tests: Although corrosion fatigue (CF) differs from SCC, many experiments apply cyclic loading in corrosive environments to see the synergy. Typically, a rotating-beam or cyclic bend test in salt solution will reveal if repeated stress fluctuations facilitate crack initiation earlier than in static SCC or pure fatigue. If a material shows dramatically shorter fatigue life in a corrosive environment than in air (with the fracture surface showing corrosion pits at initiation sites), it indicates susceptibility to corrosion-assisted cracking. These tests help determine if a component that sees vibrations or cyclic pressure might fail due to combined corrosion fatigue mechanisms.

Each of these test types has benefits and limitations, which are summarized in Table 6. Constant load and bent-beam tests directly simulate sustained stress conditions but can require long durations; SSRT accelerates time to failure but imposes a continuously increasing stress state that might not exactly replicate service; cyclic tests address conditions (like vibration) that SCC tests might omit. Oftentimes, multiple tests are used in combination to fully characterize an alloy’s behavior.

Stress Field Influence: Many SCC testing methods listed create a gradient stress field in the test specimen, primarily the U-bend and 4-point bend tests. Crack tip driving forces in this test geometry are not constant and change with position and crack depth. In testing environments with hydrogen, the gradient stress field influences the hydrogen transport rate, which can influence the effects of hydrogen-assisted cracking. Gradient stress fields can introduce synergy between SCC mechanisms, changing the required interpretation of the experimental results [69,70]. Geometry with a gradient stress field can be useful to simulate real-world applications where the expected deformation may be similar, making informed analysis of the test method used important.

### 6.2. Microscopy and Fracture Analysis

Once a test specimen has failed or cracks have formed, detailed examination of the cracks is crucial to identify the SCC mechanism. Investigators use optical microscopy for an initial overview and scanning electron microscopy (SEM) for high-resolution imaging of fracture surfaces and crack cross-sections. SEM fractography can distinguish, for example, intergranular cracking (which shows failure along grain outlines) from transgranular cleavage (which shows smooth facets within grains) or from ductile dimple rupture (which would suggest more of a corrosion fatigue failure). For SCC, branching cracks and perhaps corrosion deposits inside the cracks are common. SEM energy-dispersive X-ray spectroscopy (EDS) might detect chloride or sulfur on the fracture surface, indicating the corrosive agent.

Higher magnification methods can probe the nano-scale details; transmission electron microscopy (TEM) and atom probe tomography (APT) have been used on focused ion beam (FIB) lift-outs from crack tip regions [71]. These techniques reveal features like hydrogen traps, precipitate dissolution, or element segregation at the crack tip. For instance, APT on a 7xxx aluminum alloy SCC crack showed lithium and copper redistribution in the fracture process zone, indicating how precipitates had dissolved ahead of the crack. Such analyses confirm whether hydrogen was present (by detecting hydrides and low cohesive strength phases) or whether specific elements (like Zn or Mg in Al alloys) were selectively leached out by corrosion ahead of the crack.

Another valuable tool is electron backscatter diffraction (EBSD) in the SEM, which maps grain orientations and can show how a crack propagated relative to the microstructure. EBSD on a cracked sample might reveal, for example, that cracks in a Ni-based alloy went along high-angle grain boundaries where carbides had formed. This can support a grain-boundary embrittlement mechanism. EBSD can also identify any strain or texture changes near the crack (by examining pattern quality or shifts [72].

Chemical analysis of corrosion products in and around cracks helps to identify the failure mechanism. Techniques like EDS and WDS (wavelength dispersive spectroscopy) or secondary ion mass spectrometry (SIMS) can detect species like chloride, sulfur, or phosphorus in cracks. If one finds a high concentration of sulfide in an intergranular crack in stainless steel, it suggests that H_2_S played a role, identifying the failure as sulfide stress cracking. Raman spectroscopy or X-ray photoelectron spectroscopy (XPS) can identify the compounds present on crack surfaces (e.g., Fe_3_O_4_ vs. FeOOH oxides, or chromium oxide vs. chromium hydroxide). These clues inform whether a crack grew under active dissolution or primarily by a mechanical fracture mechanism.

Fractographic features such as striations, secondary micro-cracks, and voids provide additional evidence. Corrosion fatigue cracks often show striations from the cyclic stress, whereas SCC in the absence of cyclic load might show arrested crack “thumbnail” marks or brittle facets without striations. In hydrogen-assisted cracking, one might see quasi-cleavage and localized plastic tearing (HELP) or very flat facets (HEDE). By carefully correlating these features with the environmental history of the sample (from the test or service records), investigators deduce which SCC mechanism was dominant.

### 6.3. In Situ Monitoring Techniques

Beyond post-mortem analysis, modern approaches allow the observation or detection of SCC as it occurs. Electrochemical monitoring is widely used during SCC tests, employing a potentiostat to measure open-circuit potential (OCP), polarization resistance, or perform periodic electrochemical impedance spectroscopy (EIS), which can indicate when a crack initiates by changes in the metal’s electrochemical response [29]. For instance, a drop in polarization resistance during a slow strain rate test might signal the formation of a new crack (increasing the exposed surface area). EIS can pick up the development of new low-frequency time constants that correspond to crack propagation processes. Liu et al. [66] combined EIS with SSRT on a sensitized 5083 Al alloy and observed characteristic impedance changes correlating with SCC propagation.

Localized electrochemical techniques like the scanning vibrating electrode technique (SVET) map micro-scale anodic and cathodic activity around a crack in real time. SVET can show, for example, intense anodic current at a crack tip and cathodic currents on either side, which is proof of an active dissolution process at the crack. Similarly, tiny micro-reference electrodes placed near a crack mouth can track local solution pH or potential changes as the crack grows. A drop in pH at the crack mouth (detected by a micro-pH probe) might indicate acidification due to hydrolysis of metal ions, which in turn drives further corrosion [73].

**Figure 7:**
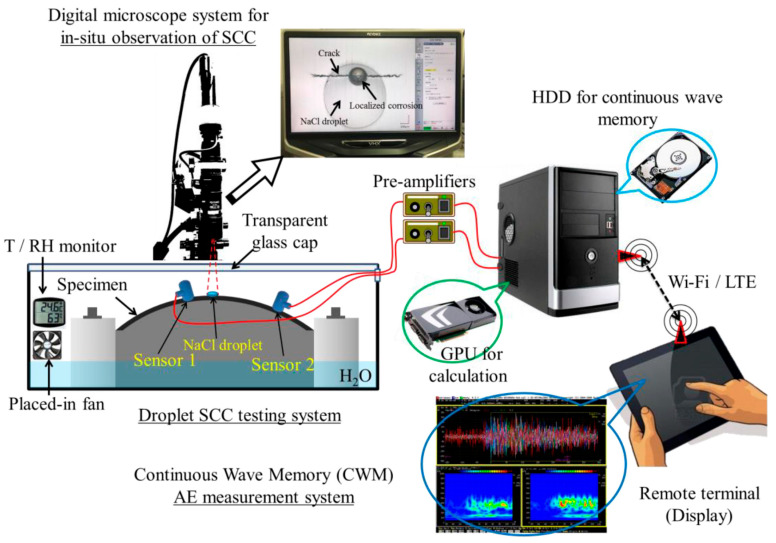
Experimental setup of in situ SCC using acoustic emission. Reproduced from [74], Materials, MDPI 2019.

Acoustic emission (AE) monitoring is a powerful in situ method. As cracks form and grow, they emit short bursts of ultrasonic noise (elastic waves) that can be detected by piezoelectric sensors on the specimen’s surface. By analyzing the rate and amplitude of acoustic emission events, researchers can often detect the moment of SCC crack initiation and monitor its propagation, Figure 7 shows a diagram of an experimental setup used for AE testing. For example, in chloride-induced SCC tests on high-strength steel, a sudden increase in AE hits coincided with crack initiation and growth, even when the crack was too small to see otherwise. AE can also help distinguish SCC from other processes. Plastic deformation tends to produce different acoustic signatures than brittle crack advances [74].

**Table 6:** Common SCC testing methods.

Method	Key Features	Advantages/Use	Limitations	References
Constant Load Test	Specimen held at fixed tensile stress in corrosive medium; observe if and when cracking or failure occurs	Closely simulates long-term service conditions; yields quantitative failure data (time-to-failure, threshold stress)	Very long test duration (for corrosion-resistant alloys, may take months/years); requires precise load maintenance	[65]
Slow Strain Rate Test (SSRT)	Tensile specimen pulled at extremely low strain rate in environment; compare ductility and fracture surface to inert environment tests	Relatively fast screening (hours to days); sensitive to SCC, clearly reveals reduced elongation or strength due to environment	Non-service loading mode (constant straining instead of static/cyclic load); results can depend on chosen strain rate (too fast may miss SCC, too slow can be overly severe)	[66]
U-Bend/C-Ring Test	Coupons bent or C-shaped to impose high tensile stress on inner/outer surface, per ASTM G30	Simple, inexpensive; can expose many samples at once for go/no-go assessment	Stress is highly non-uniform and often above yield; only provides qualitative results (cracked vs. uncracked)	[67,68]
Four-Point Bend Test	Beam loaded in four-point bending to create a uniform central moment while in environment	More uniform stress on a larger area; can obtain crack growth rate data under known stress intensity; useful for quantitative SCC propagation studies	More complex setup; bending fixture needed; not as rapid as U-bend for crack initiation (lower stress relative to yield)	[15,75]
Electrochemical Monitoring (OCP, PDP, EIS)	Track open-circuit potential, polarization curves, or impedance of specimen during SCC test	Detects crack initiation and growth in real time via changes in current or impedance; can differentiate active dissolution vs. passive behavior	Requires stable reference electrode/contacts in test; data interpretation can be complex (need to link electrochemical signals to crack events)	[29]

## 7. Engineering Strategies for SCC Mitigation

Mitigating SCC requires a multi-faceted approach that addresses the material, stress state, surface condition, and environment. Effective SCC prevention in design and maintenance involves selecting resistant materials, reducing tensile stresses, applying protective surface treatments or coatings, and controlling the service environment or adding inhibitors.

### 7.1. Material Selection and Alloy Design

The choice of base alloy is a fundamental defense against SCC. Whenever possible, engineers opt for materials that have inherently low susceptibility in the given environment. Alloys that form stable passive films and resist hydrogen uptake are preferred. For example, in marine applications, duplex or super-austenitic stainless steels (with high Mo and N) are chosen over standard grades because of their improved chloride SCC resistance. In high-temperature sour gas service, nickel-based alloys or corrosion-resistant claddings are used to avoid sulfide cracking of low-alloy steels. Materials scientists also tailor alloy chemistry to mitigate SCC; adding small amounts of elements like Nb, Ti, or Mo can stabilize grain boundaries or trap hydrogen. For instance, modern 7xxx aluminum alloys include Zr to form fine precipitates that prevent continuous grain-boundary precipitates, thus increasing SCC resistance compared to older alloys. Similarly, advanced high-strength steels sometimes incorporate controlled amounts of Ti or V to tie up C and N as carbides/nitrides, thereby preventing chromium depletion or facilitating hydrogen trapping away from critical sites [76].

Newer approaches include designing HEAs or other complex alloys specifically for aggressive settings. These alloys can form robust oxide films and distribute stress and defect sites in such a way as to delay crack initiation. Though still experimental, some HEAs have shown promise, remaining passive in boiling NaCl, where conventional alloys pit and crack. Additionally, additive manufacturing (AM) techniques allow the creation of compositionally graded materials or fine-tuned microstructures (via heat treatment of AM parts) to enhance SCC resistance where needed. For example, in a recent study, AM was used to produce a 316L stainless component with compressive residual stress and refined sub-cells, which improved its pitting and cracking resistance [77]. This process involved using a range of laser scanning patterns and analyzing the resulting microstructure using EBSD to analyze the melt pools and density with different parameters. For each parameter set, the sample was assessed for corrosion potential using potentiodynamic polarization. In summary, selecting an alloy that is “fit for environment” and, if necessary, modifying its chemistry or processing for maximum passivity/hydrogen resistance is a first line of defense against SCC.

### 7.2. Stress Reduction

Since tensile stress is required for SCC initiation, relieving or reducing these stresses can greatly diminish SCC risk. This can be done at both the design stage and after fabrication. During design, engineers avoid sharp corners or sudden cross-section changes that concentrate stress by using large filets and smooth transitions. They may also specify thicker sections or reinforcement in known high-stress areas to lower the working stress on the material. After fabrication, stress relief heat treatments, like post-weld heat treatment (PWHT) for welded pressure vessels, are commonly applied. For example, welded pipeline sections in sour service are often PWHTed to temper the martensite in the heat-affected zone and relieve residual tensile stresses, significantly reducing SCC susceptibility. There are additional methods that target surface stress, such as shot peening, laser peening, or ultrasonic peening, which impart compressive residual stress to the surface layer of a component [78,79,80]. This is highly effective because most SCC cracks start at the surface, where a compressive stress counteracts the applied tensile load. For instance, shot peening of aircraft aluminum alloy panels has been shown to practically eliminate service SCC by encasing the surface in compression. Another example is using low-plasticity burnishing or deep rolling on turbine blades to mitigate water SCC. In design, if a component cannot avoid a corrosive environment, engineers will often derate the allowable tensile stress (applying a “stress factor”) so the material operates at a fraction of its yield strength, thereby providing a margin against SCC initiation.

### 7.3. Surface Modification and Coatings

Since SCC initiates at the surface, improving the surface can prevent or delay cracks. One approach is surface machining or polishing to remove stress concentrators like scratches or tool marks. A smoother surface has fewer sites for pitting and hence for SCC to start. Surface compressive treatments (as mentioned in Stress Reduction), like laser shock peening, not only introduce compression but can also heal near-surface micro-defects by slight remelting or plastic flow.

Protective coatings are widely used as a barrier between the metal and the environment. Paints and polymer coatings like epoxy and polyurethane are common on structures and pipelines, isolating the metal from corrosive species [79,81]. For coatings to effectively prevent SCC, they must adhere well and remain intact in service so that cracks in the coating do not develop under stress. If a coating is damaged, the underlying material can experience localized attack, leading to SCC. This has driven the development of smart coatings with self-healing abilities. For example, researchers have incorporated micro-encapsulated inhibitors in coatings. If a crack forms in the coating, the capsules rupture and release corrosion inhibitors or polymerizable agents that fill the crack. Kartsonakis et al. [82] demonstrated a coating for aluminum where broken nano-capsules released a resin that re-sealed minor cracks, thus maintaining protection and drastically reducing SCC initiation sites. Other smart coatings release water-repellent or buffering compounds to locally raise pH when the coating is breached. While these advanced coatings are mostly in research phases, some are moving toward practical use on high-value structures (military aircraft components).

For more severe environments, metallic overlays or claddings are used. For example, cladding is used on the interior of a steel reactor vessel with a thin layer of Inconel alloy, which provides a corrosion-resistant (and SCC-resistant) surface, shielding the bulk steel from the environment. Thermal spray coatings (such as aluminum or zinc sprayed onto steel) act as sacrificial anodes and barriers, delaying SCC by preferentially corroding or simply blocking the environment. These, however, must be applied carefully to avoid introducing new issues like residual stresses or galvanic coupling at coating boundaries. Similarly, cold spray coatings can prevent pitting corrosion and minimize SCC potential with lower residual thermal stresses [83]. Anodizing is another surface treatment (especially for valve metals like Al and Ti) which thickens the oxide layer, making it more protective and locking in compressive stress, thereby enhancing SCC resistance for aluminum components in corrosive service. In summary, surface treatments and coatings act as frontline defenders; by eliminating surface defects, imposing surface compression, and creating a barrier, they significantly raise the threshold for SCC initiation.

### 7.4. Environmental Control and Inhibitors

Controlling the environment that a component interacts with can be one of the most effective and practical ways to prevent SCC. This includes modifying the chemistry of the fluid in contact with the material and using corrosion inhibitors or cathodic protection. A primary strategy is to remove aggressive species from the environment. For example, in boiler systems and nuclear reactors, chloride ion levels are kept extremely low (often <0.1 ppm) because even tiny chloride concentrations can drastically reduce the time to SCC in stainless steels and nickel alloys [84]. If chlorides cannot be eliminated, they might be replaced or countered by adding inhibitors like nitrate, which can sometimes counteract chlorides’ effects by shifting the corrosion potential. In oil and gas production, scavengers are injected to remove dissolved O_2_ and H_2_S from the produced fluids, thereby preventing conditions that cause sulfide SCC of steels. Oxygen control is critical in power plant feedwater. Maintaining a low dissolved oxygen, by vacuum de-aeration or chemical scavengers like hydrazine, keeps conditions reducing, so alloys like Alloy 600 stay in their immune regime and do not crack. Similarly, buffering the pH to neutral or slightly alkaline by using additives such as borates in reactor water prevents local acidification in cracks and reduces anodic dissolution rates.

When the environment itself cannot be changed, for instance, in process streams in a chemical plant, corrosion inhibitors are often used. Inhibitors are chemicals that, even in small concentrations, significantly slow corrosion reactions. Anodic inhibitors (like chromates, nitrites, molybdates) work by reinforcing or rebuilding the passive film on the metal, thus raising the threshold for SCC initiation. Cathodic inhibitors like organic amines or thioureas work by hindering the reduction reactions, like hydrogen evolution or oxygen reduction, thereby reducing the driving force for metal dissolution and hydrogen entry. Many practical inhibitor formulations combine both types and include filming agents that adsorb to form a protective layer on the metal surface. For example, phosphate-based inhibitors in cooling water lay down a protective scale on steel that prevents both general corrosion and SCC. Environmental and safety regulations have pushed for “green” inhibitors, plant extract or biodegradable inhibitors that can replace traditional ones like hexavalent chromates (which are highly effective but toxic). Recent studies show that some eco-friendly inhibitors (e.g., amino acid-based compounds) can reduce the SCC susceptibility of stainless steel almost as much as chromate, without the toxicity.

The effectiveness of inhibitors often involves their adsorption onto the metal surface. Molecules containing nitrogen, oxygen, or sulfur tend to adsorb strongly (via those heteroatoms) on the metal, blocking active sites and excluding water and aggressive ions. For instance, an inhibitor with polar functional groups may form a compact layer over a steel surface, essentially taking the place of chlorides and preventing them from reaching the metal. This lowers the corrosion current and raises the potential at which pitting or cracking would occur [85].

In closed systems, environmental control can also mean maintaining certain potentials or applying CP by either the sacrificial anode or impressed current. CP is widely used in maritime and underground structures to prevent general corrosion and SCC by keeping the metal polarized cathodically. However, CP must be applied judiciously, as excessive cathodic polarization (too negative potential) can produce excess hydrogen on the metal and lead to hydrogen-induced SCC, especially in high-strength steels. Standards such as ISO 15589-1 give potential windows that effectively mitigate corrosion without causing hydrogen embrittlement. For example, for offshore pipelines, the CP potential might be maintained around −0.8 V_SCE; this is enough to stop anodic dissolution and thus SCC by dissolution, but not so negative as to cause significant hydrogen entry.

In summary, controlling the environment involves minimizing harmful ions, keeping the solution chemistry benign, and using inhibitors or CP to suppress corrosion reactions. In practice, multiple strategies are layered. For instance, a pipeline will have a coating, CP, and possibly inhibitors injected in its contents to ensure that even if one barrier fails, others will prevent SCC. Table 5 concisely lists these mitigation methods, their mechanisms, and considerations. Table 7 summarizes the mechanisms, examples, and considerations for each SCC mitigation strategy.

In many cases, a combination of these strategies is employed for critical components. For example, a high-pressure steam pipeline may use a low-S sulfur-grade stainless steel, then stress-relieve it after fabrication, have its interior surfaces electropolished and coated, and the feedwater will be treated with oxygen scavengers and pH buffers, altogether drastically minimizing the chances of SCC. Proper selection and layering of defenses acknowledge that nothing can be made completely immune, so even if one barrier fails, others will protect the system.

Cathodic protection (CP), mentioned above, deserves special note, as it is extremely effective in preventing anodic dissolution-type SCC by shifting the metal to a corrosion potential where it does not dissolve. However, if applied incorrectly, it can cause hydrogen embrittlement. Recent studies on duplex stainless steel under CP found that cracking increased when the potential was made excessively negative due to hydrogen effects. Thus, standards provide safe cathodic potential ranges to mitigate SCC without inducing hydrogen damage [87].

## 8. Case Studies of SCC Failures in Engineering Applications

### 8.1. Oil and Gas Pipelines

A well-documented example is the 16 February 2024 rupture of a 36-inch natural gas pipeline in Canada. Investigation by the Transportation Safety Board found axial cracks consistent with near-neutral pH SCC, with the crack initiating on the external surface, where the protective coating had detached, allowing groundwater to contact the steel. Over 40 years, a colony of interlinked cracks grew longitudinally until the pipe eventually ruptured. The section had been inspected multiple times, starting in 1997 with a magnetic flux leakage tool, then most recently in 2022 with an EMAT (electromagnetic acoustic transducer) tool showing a maximum feature depth of 1–2 mm, which was below the reporting threshold [88]. Laboratory tests on vintage X52 pipeline steel from the same line under similar soil conditions confirmed that near-neutral pH SCC can grow under fluctuating pressures and soil stress. A full-scale test setup for an X52 pipeline segment where researchers applied cyclic pressure to quantify SCC growth rates was used [89]. These efforts have guided pipeline operators to implement integrity management programs, periodic hydrostatic testing (to pressure beyond operating levels to pop any incipient SCC leaks), improved coatings, and cathodic protection maintenance.

Interestingly, pipelines also suffer circumferential SCC (C-SCC) in some cases, often associated with pipe bends or significant residual stresses [57]. For example, changes in soil support (slope instability) can impart stresses on a buried pipe. In a March 2022 rupture in Illinois, a crude oil pipeline failed at a girth weld; it was determined that ground movement imposed extra strain on a less-than-perfect weld, leading to SCC at the weld fusion line of the X46 grade steel pipeline [90]. According to the report, the maximum horizontal deviation from straight was 8.2 feet, leading to an estimated bending strain of 0.42% near the girth weld. This case highlighted that SCC can be driven by external factors like soil subsidence and underscored the importance of geotechnical monitoring along pipeline routes. The mitigation involved more frequent strain monitoring of pipelines in unstable soil and ensuring weld quality and post-weld stress relief in replacements.

Modern computational tools are aiding pipeline SCC management. Finite element analysis (FEA) combined with data-driven approaches (like machine learning) is used to predict where SCC is most likely to occur by analyzing temperature, pressure cycles, soil chemistry, and pipe material data. These predictive models help prioritize inspection sites, thus preventing failures like the ones mentioned [91].

### 8.2. Nuclear Reactors and Power Plants

Nuclear power systems present a near-perfect storm for SCC, with high temperatures, water chemistry, radiation, and stress. A classic issue was stress corrosion cracking in Inconel 600 steam generator tubing and reactor vessel head penetrations in pressurized water reactors (PWRs). The primary water (high-purity water with boric acid, ~325 °C) caused intergranular SCC in Alloy 600 after years of operation. Research found that minor impurities and the metallurgical condition (grain-boundary carbides in Alloy 600) were factors. The solution was to switch to Alloy 690 (with ~30% Cr and controlled Ti content), which is far more resistant to PWSCC. Additionally, strict water chemistry control (maintaining a slightly reducing environment with dissolved hydrogen and keeping chlorides < 0.2 ppm) was implemented. Another area is boiling water reactor (BWR) internals, 304 stainless steel in oxygenated high-temperature water, experiencing irradiation-assisted SCC (IASCC). Studies show that radiation can create alloy segregation (Si or P at grain boundaries) that, combined with oxidizing water, leads to cracking [92]. Remedies include using materials like 316NG (nuclear grade) with low-carbon content (<0.03%), which results in low carbide precipitation during welding, maximizing corrosion resistance. The addition of hydrogen water chemistry (HWC) to BWRs reduces free oxygen levels and electrochemical potential, directly reducing crack growth rates in experimental testing [93,94].

New reactor concepts like small modular reactors and molten salt reactors introduce different environments like molten chloride or fluoride salts at very high temperatures (500–800 °C). These salts are extremely corrosive and can cause rapid intergranular attack and SCC in conventional alloys. In these cases, nickel superalloys or advanced stainless steels with carefully controlled grain-boundary chemistry are being explored. For example, Hastelloy N (a Ni-Mo alloy) was developed for molten fluoride salt service and includes carbide formers to pin grain boundaries and improve its resistance to embrittlement and SCC in that environment. Also, since radiation can enhance corrosion via the creation of defects, research is ongoing to tailor alloys by adding oversized solute atoms to be more radiation-tolerant and thus maintain their SCC resistance even after significant neutron exposure [95,96]. In summary, the nuclear industry responded to SCC failures by upgrading materials, modifying water chemistry through oxygen and hydrogen control, and closely monitoring critical welds and components via non-destructive examination. These steps have largely mitigated the kind of SCC that caused notorious issues in the past.

### 8.3. Aerospace Structures

**Figure 8:**
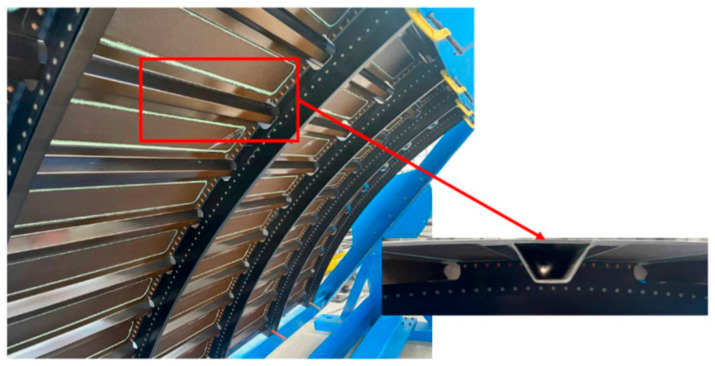
Diagram of stringer assembly in aircraft fuselage. Reproduced from [76], Materials, MDPI, 2022.

Aircraft components experience cyclic stresses and often operate in environments that can introduce moisture or salt, making SCC a concern, especially for high-strength aluminum alloys. A recent example in 2023 involved Boeing 747-8 aircraft, where certain fuselage stringer assemblies were found to have SCC due to a production shimming issue [4]. Improper shimming created gaps that led to localized flexing and paint cracking, allowing corrosion to start. Over time, cracks developed in the stringers. Figure 8 shows an image of a fuselage stringer assembly. Boeing addressed this with an airworthiness directive requiring inspections and repairs of those areas. This case underscores that manufacturing details (shimming, fastening technique) can introduce latent SCC risks even in well-chosen alloys.

SCC can occur in high-strength aerospace fasteners. High-lock nuts made of 7075-T6 aluminum, used to secure parts on an aircraft, were found to crack after as little as 2–3 years of service in humid, marine atmospheres. Analysis showed that crimping during installation induced residual tensile stresses in the nut collar. Crimping is performed with a three-jaw clamping tool, deforming the nut and increasing the clamping force of the joint, similar to a rivet. The deformation, combined with increased clamping pressure, introduces multiaxial stresses, risk of fretting, and contact pressure stresses. When exposed to a salt fog environment (during coastal operations), these stresses, combined with localized corrosion, led to intergranular SCC in the aluminum nut. After analysis, the decision was made to redesign the nut with a slightly thicker wall and use a different heat treatment to reduce residual stress and to ensure a protective oxide layer developed on the nut. Increasing the wall thickness reduced the stress and essentially eliminated the problem in subsequent tests [97].

Aerospace designers also rely heavily on protective coatings (primers and topcoats) and corrosion inhibitors in those coatings to prevent SCC on airframes. For example, many aluminum airframe parts are clad with pure aluminum or coated with chromate primers. If the top layer is scratched, the chromated primer provides temporary protection and leaches corrosion-inhibiting chromate to stop pit formation. This has successfully prevented many SCC incidents in service [98].

Spacecrafts face SCC concerns as well. The propellant tanks of rockets, often made from Al-Li alloys, are filled with oxidizers like N_2_O_4_ (nitrogen tetroxide) [7]. N_2_O_4_ can form nitric acid in the presence of moisture and under high pressure can cause SCC of the tank if not properly managed. Meticulous cleaning and humidity control are used to avoid introducing moisture that could create those conditions.

### 8.4. Marine Environments

Marine structures like ships, offshore platforms, and coastal power plants combine a chloride-rich environment with often cyclic loading from waves, tides, and differential aeration, making them prime candidates for SCC if not properly protected. Common materials like 304/316 stainless steel can crack in warm seawater under tensile stress. For instance, stainless steel tie rods in a suspension bridge over a bay might experience SCC after years due to salt spray and constant load [25]. To combat this, more SCC-resistant alloys (duplex stainless, bronze, etc.) or protective wrapping and CP are employed.

A specific example is the dry storage canisters for spent nuclear fuel at coastal sites. These are often made of 304L or 316L stainless steel and sit in the air where sea-salt particles deposit on them. Over the years, susceptible conditions (salt deposition, heat, and tensile stresses from welding) have led to chloride SCC in some of these canisters. Studies and field inspections noted that cracks tended to initiate in the weld heat-affected zones where tensile residual stresses were highest. Some facilities have since implemented periodic wash-downs to remove salt and have considered laser peening the weld areas to induce compressive stress and avoid SCC.

Cathodic protection in marine contexts must be carefully controlled to avoid hydrogen issues (as mentioned earlier with duplex stainless). Investigations on duplex S32760 steel in seawater found that if it was cathodically polarized too strongly, the failure mode shifted from traditional chloride SCC to hydrogen-induced cracking [84]. This demonstrates that even in marine environments, a balanced protection strategy (optimal potential and/or use of added inhibitors like ferrous ions or nitrite in ballast water) is required to prevent SCC.

## 9. Emerging Trends and Future Perspectives

### 9.1. High-Entropy Alloys and Advanced Materials for SCC Resistance

HEAs are drawing interest for use in aggressive environments. The complex chemistry of HEAs can form extremely robust passive oxide films and create trap sites for hydrogen, potentially making them less susceptible to SCC. Recent reviews in 2024 and 2025 have noted that many HEAs exhibit corrosion resistance on par with or better than conventional stainless steels in chloride solutions [45]. For example, an equimolar Cr-Mn-Fe-Co-Ni HEA (which forms an FCC solid solution) showed higher pitting potential and lower passive current in NaCl than 316L stainless, suggesting that it could also endure chloride exposure without cracking longer [99,100]. The reason is attributed to multi-element oxides that are more defect-tolerant; if one element’s oxide breaks down, another element’s oxide in the film continues protection.

Another emerging material strategy is hierarchically engineered microstructures. Producers of duplex stainless steel have tweaked heat treatments to obtain a fine σ-phase precipitation that, while typically unwanted, in specific distributions might block grain-boundary corrosion in some cases (this is speculative and under study). Concretely, AM techniques allow for location-specific property tuning. A component could be printed such that surfaces have one composition (for corrosion resistance) and the core another (for strength). A 2025 study showed that by altering the scan strategy in laser powder-bed fusion, they produced a 316L stainless steel with a unique sub-cell structure that significantly improved its resistance to pitting and presumably SCC [45,100].

Microstructure control is key to SCC, as continuous pathways for crack propagation should be avoided. Grain refinement, grain-boundary engineering (introducing special low-Σ boundaries), and precipitate control can all hinder SCC. A 2025 paper on duplex stainless steel [101] found that a certain heat treatment that induced some secondary phase actually raised SCC resistance in hydrogenated water by preventing localized slip, emphasizing that non-traditional microstructures might help in some environments [43,77].

In summary, advanced alloys like HEAs and other compositionally complex alloys, along with advanced processing of known alloys like AM with heat treatment, are converging toward materials that either passivate strongly or tolerate stress and hydrogen better. These are likely to find use in extreme environments like deep-sea mining, geothermal wells, and next-gen reactors. The ongoing challenge is to ensure that these materials also meet mechanical requirements and are cost-effective.

### 9.2. Application of Machine Learning in SCC Prediction and Monitoring

The rise in machine learning (ML) provides powerful new tools to handle the complexity of SCC data. There are three main avenues in which ML contributes.

(a) Interpretable Risk Models for Decision Support:

Engineers are using Bayesian networks and other probabilistic models to incorporate many variables (material, environment, stress, inspection data) and output an SCC risk level [76]. For example, in sour gas wells, a Bayesian model might consider chloride content, H_2_S partial pressure, steel sulfur content, and temperature to advise whether current conditions are within a safe domain or if SCC is likely. These models are valuable for “what-if” analysis and for ensuring that mitigation measures (like inhibitor injection) keep conditions in a low-risk range. They make the complex interactions of SCC more understandable to decision-makers by providing a structured way to see how changing one factor affects the overall risk.

(b) Supervised Learning for Prediction of Growth Rates and Susceptibility:

With enough lab and field data, ML algorithms like neural networks, support vector machines, or ensemble methods (XGBoost) can be trained to predict outcomes like crack growth rate or time to failure under certain conditions. A 2024 study [102] used XGBoost to predict SCC crack growth rate in a pipeline steel; the model not only outperformed classical empirical formulas but also identified the top factors (stress intensity, chloride concentration, temperature) influencing the crack growth. Similar classification models have been used on corroded pipeline data with SHAP (Shapley additive explanations) values to highlight why a certain section is high risk [103,104,105]. These insights can justify maintenance actions by pointing to specific evidence in the data, increasing trust in ML recommendations.

(c) Integrated Data Domain Frameworks for Asset Management:

The most promising approach is combining ML with mechanistic models like FEA or phase-field simulations and continuously monitoring data to create digital twins of infrastructure. For instance, in a pipeline, one can feed real-time sensor data, CP potential, pH, and strain from fiber optic sensors into an ML model that has been trained on both simulation and historical failure data to predict SCC initiation before it happens. A 2025 review [106] on submarine pipeline corrosion modeling concluded that ML yields the best results when it is constrained or informed by physics-based knowledge, for instance, using known electrochemical thresholds as features or using model outputs to augment sparse field data. In practical terms, this means that an operator could have a dashboard that warns, “Zone X of the pipeline likely has developed pits that could turn into SCC within Y months,” derived from an ML model that continuously learns from inspection updates and sensor readings.

ML is also expedient in materials development for corrosion resistance. There are efforts to use ML to predict which alloy compositions or heat treatments will be most SCC-resistant, by training on databases of corrosion test results. For example, a model might learn the complex relationship between composition, heat treatment, and SCC susceptibility for 7000-series aluminum and suggest new alloying strategies.

Overall, ML is making SCC management more proactive. Instead of waiting for cracks to be found in inspections, companies are trying to predict where and when they will occur and intervene beforehand. The key is integrating multi-source data, which ML excels at, and doing so in an interpretable way so that engineers trust the predictions.

### 9.3. Advanced In Situ Characterization and Simulation Approaches

Cutting-edge experiments and modeling techniques are enabling unprecedented insight into SCC mechanisms and are likely to drive future prevention strategies. Non-destructive in situ X-ray tomography using synchrotron sources allows researchers to watch cracks initiate and grow inside a metal sample in real time. A 2024 study [107] performed real-time 4D imaging of SCC in a sensitized stainless steel, capturing the nucleation of intergranular cracks from small pits and seeing how they linked up over time. These observations provide direct validation for theories like film rupture or hydrogen-assisted cracking by showing branching patterns or transitions in crack modes. As synchrotron micro-CT becomes more accessible, more SCC phenomena (especially in lab coupons) will be documented this way, yielding data on crack growth rates and morphologies that can feed into models.

Phase-field modeling has emerged as a powerful simulation tool for fracture and corrosion [108]. It can simulate crack initiation and propagation by coupling chemical free energy, electrochemical reactions, and mechanical forces in a unified framework. Recent phase-field models have been able to reproduce the dendritic crack patterns seen in stainless steels undergoing chloride SCC, and even capture the mixed mode where hydrogen embrittlement assists crack growth at the tip [109]. For example, a 2025 study [110] developed a chemo-mechanical phase-field model that included both dissolution and hydrogen effects to simulate SCC in a pipeline steel under cathodic protection [111]. The model showed how over-polarization lowered the fracture energy and switched the crack path from one governed by dissolution to one dominated by hydrogen embrittlement, aligning with experimental findings in subsea pipelines.

A notable simulation advancement is microstructure-sensitive modeling. Grain-scale models incorporate actual grain orientation data from EBSD to see how a crack might deflect or stop at certain grain boundaries. These help predict whether a certain texture, like rolling direction, will encourage transgranular cracking along easy slip planes. One 2025 model used real 3D polycrystalline structures and successfully predicted crack branching in a textured alloy, matching what is seen in experiments [110].

On the experimental side, integrations like acoustic emission and electrochemistry are being attempted to get a holistic view of SCC as it unfolds, capturing mechanical, chemical, and electrical signals together. These comprehensive data sets are ideal for training the ML models, closing the loop between understanding and prediction.

In terms of materials development, advanced characterization and simulation allow for designing out SCC. With tools like APT revealing exactly which impurities at grain boundaries cause embrittlement, metallurgists can adjust processing (e.g., reduce silicon in stainless steel to mitigate radiation-induced segregation, as noted by the DOE for IASCC). With phase-field models, engineers can virtually test how a proposed alloy composition might behave in each environment, before ever fabricating it, by simulating its cracking behavior.

Overall, these emerging trends, new alloys, intelligent data analysis, and sophisticated modeling strategies are converging towards a future where SCC can be predicted and prevented more reliably. Engineers may soon have digital twins of critical structures that forecast SCC risk in real time and suggest optimal interventions like adjusting inhibitor dosing or scheduling a stress-relief anneal far before a crack forms, turning SCC from a sudden threat into a manageable aspect of materials performance.

## 10. Conclusions

SCC is a multifaceted failure mode requiring both tensile stress and a corrosive environment; it cannot be treated as purely a corrosion problem or purely a mechanical problem. A combination of mechanisms (film rupture, hydrogen embrittlement, and adsorption-induced cleavage) may operate simultaneously at a crack tip. Bulk environmental measures (like overall corrosion rate or general corrosion resistance) often do not predict SCC susceptibility, because SCC is driven by localized conditions. For instance, a tiny, occluded crack tip area can become acidic or hydrogen-rich even if the bulk solution is neutral. Microstructure matters greatly, as grain size and orientation, precipitates, and residual stresses from fabrication (welding or cold work) strongly influence how easily cracks nucleate and propagate. In additive manufacturing or other novel processes, post-treatment is often needed to optimize microstructure for SCC resistance (stress relief, hot isostatic pressing to eliminate internal defects). Elevated temperature and pressure generally accelerate SCC. Designs must account for higher crack growth rates and shorter incubation times at service temperatures, which means applying stricter safety factors or using more resistant materials in those conditions. Testing for SCC should be done under service-simulating conditions (temperature, chemistry, stress mode) because room-temperature or benign-environment tests can miss vulnerabilities. Also, importantly, no single mitigation technique is foolproof. Effective SCC prevention is layered, combining good material selection, proper stress engineering, protective coatings, and environmental control. Future developments like machine learning predictive maintenance and new smart coatings or materials will further help manage SCC, but the core approach remains the same, which is to understand the stresses and environment your component will face, and address both in the design and maintenance.

A successful SCC prevention program requires coordination between design engineers, materials engineers, and corrosion specialists at all stages. First, a thorough analysis of the service environment is essential, identifying aggressive species and considering temperature and pressure extremes, the presence of crevices or deposits, and even the potential microbiological activity. With that knowledge, one should choose materials that can withstand those conditions or plan for coatings or inhibitors.

## Figures and Tables

**Table 2:** Comparison of crack growth rates and yield strength in aluminum alloys.

Alloy	Temp C	Yield Strength MPa	Crack Growth Rate	Environment	Reference
6005A	35	260–275	5.88 × 10^−7^ mm/s	3.5% NaCl	[33]
2024-T3	25	324–345	~10^−7^–10^−6^ mm/s	3.5% NaCl	[34]

**Table 3:** Material classes and their SCC susceptibility.

Material Class	SCC Susceptibility	Common Industries	Contributing Factors	References
Austenitic Stainless Steels (304, 316L, 904L)	Moderate to high in hot chloride environments; much lower in cool or de-aerated conditions.	Chemical processing, desalination, marine, power (condenser tubes).	Sensitization (chromium carbide at grain boundaries), presence of chlorides, tensile weld stresses, crevices trapping chlorides.	[1,23,24]
Duplex Stainless Steels (2205, 2507	Low to moderate; better chloride SCC resistance than austenitic up to ~120 °C. Can suffer hydrogen-induced cracking in sour service or under cathodic protection.	Offshore oil & gas (pipes, platforms), pulp & paper, desalination.	Imbalanced phase ratio (too much ferrite or austenite), high-temperature (>130 °C) chlorides, hydrogen pickup in H_2_S environments, improper welding (sigma phase formation).	[1,23,24]
High-Strength Aluminum Alloys (2xxx, 7xxx series)	High in susceptible tempers. 7xxx (e.g., 7075-T6) high SCC risk (e.g., stress-corrosion cracks in aircraft skins); 2xxx (2024-T3) moderate to high (often intergranular SCC in airframe components). 5xxx (Al-Mg like 5083) low, unless sensitized by elevated temp exposure (then high); 6xxx (6061) low to moderate. Al-Li alloys moderate or low (improved vs. older alloys).	Aerospace structures (fuselage, ribs 2xxx, 7xxx), marine (5083 in ships), automotive frames (6xxx), spacecraft/rocket propellant tanks (Al-Li).	Grain-boundary precipitates (e.g., MgZn_2_ in 7xxx, Al_2_Cu in 2xxx) which create anodic paths; environmental humidity and salt exposure; residual cold-work stress; sensitization (β phase in 5xxx).	[26,27,28,29,30,31,32]
Titanium Alloys (Ti, Ti-6Al-4V)	Low in most environments (seawater, chlorides); excellent SCC resistance. Under very specific conditions (high cathodic potentials or methanol presence), moderate via hydrogen-induced cracking.	Aerospace (airframes, fasteners), biomedical (implants), chemical processing (heat exchangers), marine (pumps, valves).	Hydrogen absorption (from cathodic protection or H_2_S) leading to hydride formation; hot bromide or chloride in the presence of stress (rare cases). Generally, Ti’s SCC risk is low unless improper cathodic protection causes hydrogen embrittlement.	[39,40,41]
Nickel-Based Alloys 600/625/718, C-276, 59	Low to very low in most corrosive media, e.g., Inconel 600 is susceptible to caustic SCC and PWR primary-water SCC over long term; C-276 is extremely resistant even in hot concentrated chlorides; Monel 400 can crack in strongly oxidizing acids but not in seawater.	Nuclear reactors and power plants (steam generator tubes Inconel 690, reactor internals), oil & gas (downhole tubing Incoloy 825, valves Monel), chemical processing (acid heaters Hastelloy).	Specific environment alloy pairings, e.g., Inconel 600 in high-purity water at 330 °C suffered intergranular SCC (addressed by switching to Alloy 690); exposure to caustic solutions (NaOH) at high temp can crack Ni alloys if passive film stability is compromised. Cold work can slightly increase susceptibility, so solution-annealed conditions are preferred for critical uses.	[37,38]
High-Entropy Alloys (HEAs)	Low (emerging) early tests show many HEAs have comparable or better SCC resistance than stainless steels, but data are limited. Compositionally complex, behavior depends on elements present.	Currently in R&D; potential use in extreme environments (marine, nuclear) and as protective coatings.	Multi-element composition yields robust passive films (if Cr/Mo present) and can trap hydrogen in stable phases, potentially reducing SCC. However, variability in microstructure (segregation, second phases) from processing can introduce vulnerabilities. Ongoing research focuses on ensuring phase stability and minimizing defects in HEAs to capitalize on their inherent SCC resistance.	[43,44,45,46]

**Table 4:** Corrosion potential of common structural materials in a 3.5% NaCl solution at standard temperature 25 °C.

Material	E_corr (V)	Reference Electrode	SCC Behavior	Reference
304 SS	−0.2 to −0.35	Ag/AgCl, KCl saturated	Chloride SCC	[48]
316SS	−0.15 to −0.30	Ag/AgCl, KCl saturated	Chloride SCC	[47]
6005 Aluminum	−0.70 to −0.80	Saturated calomel electrode (SCE)	SCC with high stresses	[49]
6061 Aluminum	−0.70 to −0.80	Ag/AgCl, KCl saturated	SCC with high stresses	[50]
High-Strength Steel (X70)	−0.60 to −0.75	Ag/AgCl, KCl saturated	Hydrogen-assisted	[51]
Ni Alloy 600	−0.10 to −0.25	Saturated calomel electrode (SCE)	SCC at higher temperatures	[52]
Commercially Pure Titanium	−0.49 to −0.52	Saturated calomel electrode (SCE)	High resistance	[53]

**Table 7:** SCC mitigation strategies, mechanisms, examples, and considerations.

Strategy	Mechanism	Examples	Considerations	References
Material Selection and Alloy Design	Choose alloys with robust passive films and low hydrogen diffusivity so they inherently resist SCC. Fine-tune chemistry to avoid deleterious phases.	Ti grade 2 in chloride water (immune to chloride SCC); 2205 duplex SS in sour water (resists cracking better than 304); add Mo, N to stainless steel for improved pitting/SCC; use post-heat-treated AM 316L with refined microstructure.	Balance material cost vs. performance; ensure chosen alloy meets mechanical needs (strength, weldability) as well as SCC resistance. Verify microstructure (no sensitization or gross segregation) especially in welds or AM parts.	[76,86]
Stress Reduction	Minimize tensile and residual stresses that drive SCC.	PWHT of welds in pressure vessels (relieves residual stress); shot-peening aircraft Al wing skins (imparts surface compression); designing filets instead of sharp corners in a crankshaft.	Full stress relief may be impractical if large components; compressive treatments must be applied uniformly and may require periodic re-application if stresses relax over time. Avoid introducing surface damage during peening.	[78,79,80]
Surface Modification and Coatings	Provide a protective barrier or sacrificial layer; eliminate surface defects.	Polishing and passivating stainless steel tubing (removes inclusions, creates a stable oxide); anodizing 7075-T6 fittings (thicker oxide to prevent SCC); thermal-sprayed Al or Zn on steel (sacrificial anode coating); smart polymer coating on aluminum with encapsulated corrosion inhibitors.	Coating must adhere well and tolerate deformation without cracking. Watch for galvanic interactions (e.g., a coating too noble can create a galvanic couple). Maintenance of coating is critical; if coating breaks down, SCC can localize at the defect.	[79,80,81,82]
Environmental Control and Inhibitors	Adjust environment to be less aggressive; use chemicals to stifle corrosion processes.	De-aeration of boiler water (add hydrazine to remove O_2_); chloride removal via water purification in a refinery cooling loop; add nitrite inhibitor in rebar concrete (reduces corrosion); cathodic protection of a buried pipeline at −0.85 V vs Ag/AgCl; injecting filming amine inhibitors in oil wells.	Over-polarization in CP can lead to hydrogen cracking and must maintain proper potential window. Inhibitors can be consumed over time or suffer from flow conditions, so require monitoring & renewal. Ensure inhibitors or water chemistry adjustments do not introduce new issues (e.g., avoid chromate inhibitors if environmental regulations forbid them; check that pH buffers do not cause other forms of corrosion).	[84]

## Data Availability

No new data were created or analyzed in this study. Data sharing is not applicable to this article.

## References

[1] Vakili M., Koutník P., Kohout J., Gholami Z. (2024). Analysis, Assessment, and Mitigation of Stress Corrosion Cracking in Austenitic Stainless Steels in the Oil and Gas Sector: A Review. Surfaces.

[2] Zhang X., Wang D., Cao F., Wang C., Zha M. (2025). Bridging static corrosion behavior and stress corrosion cracking susceptibility in dilute Mg-Zn-Ca alloys. Corros. Sci..

[3] Wanhill R.J.H., Byrnes R.T., Smith C.L. (2011). Stress corrosion cracking (SCC) in aerospace vehicles. Stress Corrosion Cracking.

[4] Federal Aviation Administration (2023). Airworthiness Directives; The Boeing Company Airplanes.

[5] Mohanty S., Majumdar S., Natesan K. (2012). A Review of Stress Corrosion Cracking/Fatigue Modeling for Light Water Reactor Cooling System Components.

[6] Ehrnstén U., Andresen L., Que Z. (2024). A review of stress corrosion cracking of austenitic stainless steels in PWR primary water. J. Nucl. Mater..

[7] Zhao Y., Tian G., Liu D., Ren B., Zhang W., Zhu Y. (2025). Evaluation of Stress Corrosion Cracking Susceptibility of 2195-T8 Al-Li Alloy in Propellant Environment Using Slow Strain Rate Testing. Aerospace.

[8] Yao Y., Quach W.-M. (2023). Numerical Study on Residual Stresses and Plastic Strains in Cold-Formed High-Strength Steel Circular Hollow Sections. Materials.

[9] Mutafi A., Irwan J., Yidris N., Alshalif A.F., Saif Y., Abdulrahman H., Mutaafi A., Al-Ashmori Y.Y., Amran M., Maureira-Carsalade N. (2025). Residual stresses in cold-formed steel sections: An overview of influences and measurement techniques. Forces Mech..

[10] Huang X., Costa D., Diawara B., Maurice V., Marcus P. (2025). Protection of Stainless Steels by Mo against Cl Attack: A DFT Study. J. Phys. Chem. C.

[11] Coelho L.B., Amand T., Torres D., Olivier M., Ustarroz J. (2025). Identifying stable pitting pathways in 316L stainless steel via fractal-inspired PCA-based clustering. npj Mater. Degrad..

[12] Trentin A., Mardoukhi A., Lambai A., Pohjanne P., Huttunen-Saarivirta E. (2025). Pitting corrosion of austenitic and duplex stainless steels in dilute acids at elevated temperature: Effect of electrolyte chemistry and material microstructure. Corros. Sci..

[13] Akid R. (2025). Corrosion fatigue—Understanding damage from smooth surfaces. Theor. Appl. Fract. Mech..

[14] Li X., Zhang J., Cui Y., Djukic M.B., Feng H., Wang Y. (2024). Review of the hydrogen embrittlement and interactions between hydrogen and microstructural interfaces in metallic alloys: Grain boundary, twin boundary, and nano-precipitate. Int. J. Hydrogen Energy.

[15] Euesden R.T., Curd M.E., Yao Y., Grant C., Holroyd N.H., Prangnell P.B., Burnett T.L. (2025). Direct comparison of the environmentally induced cracking resistance of 2nd and 3rd generation alloys, AA7050-T7651 and AA7085-T7651. Mater. Des..

[16] Hamdan H., Alsit A., Al Tahhan A.B., Mughieda O., Mourad A.-H.I., Shehadeh M.A., Alkhedher M. (2024). Prognosis methods of stress corrosion cracking under harsh environmental conditions. Heliyon.

[17] Mohtadi-Bonab M.A. (2019). Effects of Different Parameters on Initiation and Propagation of Stress Corrosion Cracks in Pipeline Steels: A Review. Metals.

[18] Javeria U., Kim S.J. (2025). Investigation of hydrogen embrittlement in steel alloys: Mechanism, factors, advanced methods and materials, applications, challenges, and future directions: A review. J. Mater. Res. Technol..

[19] Gregorovičová E., Pospíšil J. (2025). Hydrogen Safety in Energy Infrastructure: A Review. Energies.

[20] Felix L.C., Li Q.-K., Penev E.S., Yakobson B.I. (2025). Ab Initio Molecular Dynamics Insights into Stress Corrosion Cracking and Dissolution of Metal Oxides. Materials.

[21] Zhang Y., Zheng K., Heng J., Zhu J. (2019). Corrosion-Fatigue Evaluation of Uncoated Weathering Steel Bridges. Appl. Sci..

[22] Katona R.M., Taylor J.M., McCready T.A., Bryan C.R., Schaller R.F. (2024). Towards understanding stress corrosion cracking of austenitic stainless steels exposed to realistic sea salt brines. Corros. Sci..

[23] Hassan H.M. (2023). Evaluation of several austenitic varieties of stainless steel’s chemical corrosion resistance. Chem. Methodol..

[24] Reda A., Shahin M.A., Montague P. (2025). Review of Material Selection for Corrosion-Resistant Alloy Pipelines. Eng. Sci..

[25] Yeh C.-P., Tsai K.-C., Huang J.-Y. (2025). Environmental Factors Influencing Stress Corrosion Cracking Behavior of Austenitic Stainless Steels in Simulated Seawater. Materials.

[26] Wu H., Wang L., Bian G., Wei Z., Tian H., Cui Z. (2025). Determining the controlling factor of the stress corrosion cracking of 2024 aluminum alloy with different heat treatments in thin electrolyte layer environment. Mater. Charact..

[27] Charalampidou C.M., Siskou N., Georgoulis D., Kourkoulis S.K., Zheludkevich M., Alexopoulos N.D. (2024). Corrosion of aluminium alloy AA2024-Τ3 specimens subjected to different artificial ageing heat treatments. npj Mater. Degrad..

[28] Yang X., Fan W., Zhang Y. (2024). The influence Cl^−^ on stress corrosion of 7xxx series aluminium alloys studied by experimental and simulation technology. Heliyon.

[29] Kharal S., Jones N.J., Petculescu G. (2025). Sensitization of 5xxx Series Aluminum Alloys Explored with an Adapted Johnson-Mehl-Avrami-Kolmogorov Model for Phase Evolution and Ultrasonics. Corrosion.

[30] Fujii T., Endo Y., Shimamura Y. (2025). Nucleation of stress corrosion cracking in 6061 aluminum alloy in sodium chloride solution: Electrochemical aspects. J. Alloys Compd..

[31] Charalampidou C.M., Pretorius C.C., Salojee M., Karousos D., Khodja M., Mostert R.J., Alexopoulos N.D. (2025). Investigation of the mechanisms affecting corrosion susceptibility of wrought aeronautical aluminium alloys Al–Cu–Li (AA2198) and Al–Cu–Mg (AA2024) for different pre-stretching levels. J. Mater. Res. Technol..

[32] Wang T., Wen K., Lin B., Li X., Li Y., Li Z., Zhang Y., Xiong B. (2023). Influence of Cryogenic Temperatures on the Mechanical Properties and Microstructure of 2195-T8 Alloy. Metals.

[33] Ma J., Sun J., Guan Q., Yang Q., Tang J., Zou C., Wang J., Tang B., Kou H., Wang H. (2021). The Localized Corrosion and Stress Corrosion Cracking of a 6005A-T6 Extrusion Profile. Materials.

[34] Baart B.V. (2020). Stress-Corrosion Cracking Susceptibility of Polystyrene/TiO_2_ Nanocomposite Coated Thin-Sheet Aluminum Alloy 2024—T3 with 3.5% NaCl. Master’s Thesis.

[35] Blanc C., Lavelle B., Mankowski G. (1997). The role of precipitates enriched with copper on the susceptibility to pitting corrosion of the 2024 aluminium alloy. Corros. Sci..

[36] Siwowski T.W. (2009). Structural behaviour of aluminium bridge deck panels. Eng. Struct..

[37] Iqbal M.A., Skotnicová K., Shafiq A., Sindhu T.N. (2025). Inconel alloys: A comprehensive review of properties and advanced manufacturing techniques. Int. J. Thermofluids.

[38] Wang X., Wang G., Wang W., Liu X., Liu Y., Jin Y., Zhang Y. (2025). Enhancing corrosion resistance of nickel-based alloys: A review of alloying, surface treatments, and environmental effects. J. Alloys Compd..

[39] Liu R., Xie Y., Jin Y., Cui Y., Liu L., Wang F. (2023). Stress corrosion cracking of the titanium alloys under hydrostatic pressure resulting from the degradation of passive films. Acta Mater..

[40] Yang T., Wang Z., Zhang C.-H. (2025). The impact of hydrides on crack propagation in dual-phase Ti-6Al-4V alloy. J. Mater. Sci..

[41] Chen W., Wang W., Liu L., Liu R., Cui Y., Chen Z., Wang Q., Wang F. (2024). The effect of NaCl-induced corrosion on Ti60’s hot salt stress corrosion cracking. Corros. Sci..

[42] Xu Y., Zhang X., Lu X., Zhang S., Sun Z., Gao F., Li J., Lai M. (2024). Influences of microstructures and macrozones on the stress corrosion cracking sensitivity of a near alpha titanium alloy. Corros. Sci..

[43] Oketola A.M., Adegbola T.A., Jamiru T., Ogunbiyi O., Salifu S. (2025). Advances in High-Entropy Alloy Research: Unraveling Fabrication Techniques, Microstructural Transformations, and Mechanical Properties. J. Bio Tribo Corros..

[44] Varshney P., Kumar N. (2024). Early Stages of Crack Nucleation Mechanism in Fe_39_Mn_20_Co_20_Cr_15_Si_5_Al_1_ High-Entropy Alloy during Stress Corrosion Cracking Phenomenon: Pit Initiation and Growth. Crystals.

[45] Yu B., Ren Y., Zeng Y., Ma W., Morita K., Zhan S., Lei Y., Lv G., Li S., Wu J. (2024). Recent progress in high-entropy alloys: A focused review of preparation processes and properties. J. Mater. Res. Technol..

[46] Varshney P., Kumar N. (2023). Investigating the susceptibility of Fe_39_Mn_20_Co_20_Cr_15_Si_5_Al_1_ high entropy alloy to stress corrosion cracking in a 3.5 wt.% NaCl environment. Corros. Sci..

[47] Gudić S., Vrsalović L., Matošin A., Krolo J., Oguzie E.E., Nagode A. (2023). Corrosion Behavior of Stainless Steel in Seawater in the Presence of Sulfide. Appl. Sci..

[48] Zhou X.Y., Lvov S.N., Wei X.J., Benning L.G., Macdonald D.D. (2002). Quantitative evaluation of general corrosion of Type 304 stainless steel in subcritical and supercritical aqueous solutions via electrochemical noise analysis. Corros. Sci..

[49] Li W., Chen X., Chen B. (2018). Effect of aging on the corrosion behavior of 6005 Al alloys in 3.5 wt% NaCl aqueous solution. J. Mater. Res..

[50] Khalaf M.M., Salih A.-E., Al-Saadi T.H.A., Mustafa A.M. (2025). Comparative Study of Corrosion Behavior of AA6061 and AA5086 Aluminum Alloys in Polluted Local Seawater -Iraq. Corros. Sci. Technol..

[51] Giarola J.M., Calderón-Hernández J.W., Conde F.F., Marcomini J.B., de Melo H.G., Avila J.A., Filho W.W.B. (2021). Corrosion Behavior and Microstructural Characterization of Friction Stir Welded API X70 Steel. J. Mater. Eng. Perform..

[52] Łyczkowska K., Michalska J. (2017). Studies on the Corrosion Resistance of Laser-Welded Inconel 600 and Inconel 625 Nickel-Based Superalloys. Arch. Metall. Mater..

[53] Malinovschi V., Marin A.H., Ducu C., Moga S., Andrei V., Coaca E., Craciun V., Lungu M., Lungu C.P. (2021). Improvement of Mechanical and Corrosion Properties of Commercially Pure Titanium Using Alumina PEO Coatings. Coatings.

[54] Wei B., Cai Z., Niu M., Xu J., Liao B., Wu T., Yu C., Sun C. (2025). Synergistic microbial interactions and electrochemical mechanisms driving microbiologically influenced corrosion in offshore platform produced seawater at 60 °C. Corros. Sci..

[55] Liu B., Lu F., Zhu S., Du C., Li X. (2024). Enhancement resistance to microbiologically influenced stress corrosion of Cu-bearing steel against Bacillus cereus. npj Mater. Degrad..

[56] Sun Q., Xie F., Zhu M., Wang L., Wang D., Wu M. (2025). Analysis of stress corrosion cracking failure of 316L stainless steel flow promoter string in the aggressive oilfield environment. Eng. Fail. Anal..

[57] Shirazi H., Eadie R., Chen W. (2023). A review on current understanding of pipeline circumferential stress corrosion cracking in near-neutral PH environment. Eng. Fail. Anal..

[58] Martin U., Birbilis N., Macdonald D.D., Bastidas D.M. (2023). Pit-to-crack mechanisms of 316LN stainless steel reinforcement in alkaline solution influenced by strain induced martensite. npj Mater. Degrad..

[59] Galakhova A., Prattes K., Mori G. (2021). High-temperature high-pressure SCC testing of corrosion-resistant alloys. Mater. Corros..

[60] Wang Z., Xue Y., Wang R., Wu J., Zhang Y., Xue H. (2024). Review on crack growth driving force at the tip of stress corrosion cracking in the safe end dissimilar metal welded joint. Nucl. Eng. Des..

[61] Yan K., Shi B., Ma S., Zhu Z. (2025). Effect of Stress on High-Temperature Molten Salt Corrosion of T91 Steel. Metals.

[62] Bosch R.-W., Ritter S., Herbst M., Kilian R., Burke M.G., Duff J., Scenini F., Gu Y., Dinu A., Ehrnstén U. (2021). Stress corrosion crack initiation testing with tapered specimens in high-temperature water—Results of a collaborative research project. Corros. Eng. Sci. Technol..

[63] Lopez-Dominguez D., Gomez-Guzman N.B., Porcayo-Calderón J., Lopez-Sesenes R., Larios-Galvez A.K., Sarmiento-Bustos E., Rodriguez-Clemente E., Gonzalez-Rodriguez J.G. (2023). An Electrochemical Study of the Corrosion Behaviour of T91 Steel in Molten Nitrates. Metals.

[64] Faisal N.H., Rajendran V., Prathuru A., Hossain M., Muthukrishnan R., Balogun Y., Pancholi K., Hussain T., Lokachari S., Horri B.A. (2024). Thermal spray coatings for molten salt facing structural parts and enabling opportunities for thermochemical cycle electrolysis. Eng. Rep..

[65] Wang Y., Chen M., Zhao Y. (2019). Preparation and Corrosion Resistance of Microarc Oxidation-Coated Biomedical Mg–Zn–Ca Alloy in the Silicon–Phosphorus-Mixed Electrolyte. ACS Omega.

[66] Liu Z., Wang J., Qin Z., Xia D.-H., Behnamian Y., Hu W., Tribollet B. (2025). A mechanistic study on stress corrosion cracking of sensitized AA5083 in a simulated water level fluctuation zone: Combined impedance analysis and tensile tests. Corros. Sci..

[67] Fan Y., Lu Y.H., Wang F., Hong C., Shoji T. (2025). Development of a methodology to study the stress corrosion cracking behavior in high-temperature and high-pressure water environment based on small punch test. Nucl. Eng. Des..

[68] Yang J., Chen X., Wu L., Ding X., Jiao J., Cui M., Lian Y., Zhang J., Ji P., Wu S. (2025). Stress corrosion behavior using in-situ stress-electrochemical method of 7A52 aluminum alloy prepared by various processes. Corros. Sci..

[69] Nelson G., Eadie R., Chen W. (2024). The role of hydrogen embrittlement in the near-neutral pH corrosion fatigue of pipeline steels. Corros. Sci..

[70] Williams K.S., Chauhan R.R., Cooper K., Thomas N., Ahmed K., Shao L. (2025). Combining four-point bending and corrosion to map stress-dependent corrosion susceptibility of 316H stainless steel in FLiNaK. Mater. Today Commun..

[71] Chen Y.-S., Huang C., Liu P.-Y., Yen H.-W., Niu R., Burr P., Moore K.L., Martínez-Pañeda E., Atrens A., Cairney J.M. (2025). Hydrogen trapping and embrittlement in metals—A review. Int. J. Hydrogen Energy.

[72] Freixes M.L., Peguet L., Warner T., Gault B. (2024). Nanoscale perspective on the stress-corrosion cracking behavior of a peak-aged 7XXX-Al alloy. Corros. Sci..

[73] Nuthalapati S., Kee K.E., Pedapati S.R., Jumbri K. (2024). A review of chloride induced stress corrosion cracking characterization in austenitic stainless steels using acoustic emission technique. Nucl. Eng. Technol..

[74] Wu K., Ito K., Shinozaki I., Chivavibul P., Enoki M. (2019). A Comparative Study of Localized Corrosion and Stress Corrosion Cracking of 13Cr Martensitic Stainless Steel Using Acoustic Emission and X-ray Computed Tomography. Materials.

[75] Li B., Gong Y., Gao Y., Hou M., Li L. (2022). Failure Analysis of Hat-Stringer-Stiffened Aircraft Composite Panels under Four-Point Bending Loading. Materials.

[76] Zuniga A.R., Bakhtiari S., Aldrich C., Calo V.M., Iannuzzi M. (2025). Predicting stress corrosion cracking in downhole environments: A Bayesian network approach for duplex stainless steels. npj Mater. Degrad..

[77] Moazzen P., Shahriari A., Shamsdini S., Seraj P., Forooghi F., Aghayar Y., Shakerin S., Purdy M.R., Mohammadi M. (2025). Optimum corrosion performance using microstructure design and additive manufacturing process control. npj Mater. Degrad..

[78] John M., Ralls A.M., Misra M., Menezes L. (2023). Effect of ultrasonic impact peening on stress corrosion cracking resistance of austenitic stainless-steel welds for nuclear canister applications. J. Nucl. Mater..

[79] Jose S.A., John M., Misra M., Menezes L. (2025). Peening Techniques for Mitigating Chlorine-Induced Stress Corrosion Cracking of Dry Storage Canisters for Nuclear Applications. Materials.

[80] John M., Kalvala R., Misra M., Menezes L. (2021). Peening Techniques for Surface Modification: Processes, Properties, and Applications. Materials.

[81] Wei H., Wang Y., Guo J., Shen N.Z., Jiang D., Zhang X., Yan X., Zhu J., Wang Q., Shao L. (2015). Advanced micro/nanocapsules for self-healing smart anticorrosion coatings. J. Mater. Chem. A.

[82] Kartsonakis I.A., Kontiza A., Kanellopoulou I.A. (2024). Advanced Micro/Nanocapsules for Self-Healing Coatings. Appl. Sci..

[83] Daroonparvar M., Helmer A., Ralls A., Khan M.F., Kasar A., Gupta R., Misra M., Shao S., Menezes P., Shamsaei N. (2023). Pitting corrosion behavior and corrosion protection performance of cold sprayed double layered noble barrier coating on magnesium-based alloy in chloride containing solutions. J. Magnes. Alloys.

[84] Yeh C.-P., Tsai K.-C., Huang J.-Y. (2020). Influence of Chloride Concentration on Stress Corrosion Cracking and Crevice Corrosion of Austenitic Stainless Steel in Saline Environments. Materials.

[85] Răuță D.-I., Matei E., Avramescu S.-M. (2025). Recent Development of Corrosion Inhibitors: Types, Mechanisms, Electrochemical Behavior, Efficiency, and Environmental Impact. Technologies.

[86] Samaei A., Chaudhuri S. (2022). Role of Zirconium Conversion Coating in Corrosion Performance of Aluminum Alloys: An Integrated First-Principles and Multiphysics Modeling Approach. Electrochim. Acta.

[87] Pan Y., Sun B., Chen H., Liu Z., Dai W., Yang X., Yang W., Deng Y., Li X. (2024). Stress corrosion cracking behavior and mechanism of 2205 duplex stainless steel under applied polarization potentials. Corros. Sci..

[88] Transportation Safety Board of Canada (2024). Pipeline Transportation Safety Investigation Report P24H0018.

[89] Sun H., Yan L., Bibby D., Gravel J., Kang J., Zhou W. (2024). Full-scale testing of near-neutral pH stress corrosion cracking growth behavior of a vintage X52 oil pipe. Fatigue Fract. Eng. Mat. Struct..

[90] National Transportation Safety Board (2023). Marathon Pipe Line LLC Pipeline Rupture and Crude Oil Release.

[91] Sarwar U., Mokhtar A.A., Muhammad M., Wassan R.K., Soomro A.A., Wassan M.A., Kaka S. (2024). Enhancing pipeline integrity: A comprehensive review of deep learning-enabled finite element analysis for stress corrosion cracking prediction. Eng. Appl. Comput. Fluid Mech..

[92] John M., Ralls A.M., Misra M., Menezes L. (2024). Understanding the Mechanism of Stress Corrosion Cracking Resistance in Stainless Steel Welds Subjected to Laser Shock Peening without Coating for Nuclear Canister Applications. J. Mater. Eng. Perform..

[93] Du D., Song M., Chen K., Zhang L., Andresen P.L. (2020). Effect of deformation level and orientation on SCC of 316L stainless steel in simulated light water environments. J. Nucl. Mater..

[94] Was G.S., Singlawi A., Swaminathan S., Sun K. (2023). The Mechanism of Irradiation Assisted Stress Corrosion Cracks in Stainless Steels.

[95] Wei Y., La P., Zheng Y., Zhan F., Yu H., Yang P., Zhu M., Bai Z., Gao Y. (2025). Review of Molten Salt Corrosion in Stainless Steels and Superalloys. Crystals.

[96] Liu H., Lei G.-H., Huang H.-F. (2024). Review on synergistic damage effect of irradiation and corrosion on reactor structural alloys. Nucl. Sci. Tech..

[97] Cheng Q., Ye L., Wang S., Gao Q., Xu Y., Xu Y., Chen Y. (2024). Analysis of Cracking of 7075 Aluminum Alloy High-Lock Nuts. Metals.

[98] Zhu H., Li J. (2024). Advancements in corrosion protection for aerospace aluminum alloys through surface treatment. Int. J. Electrochem. Sci..

[99] Liang J., Wang W., Cao Z., Guo J., Sun Z., Hai Y. (2024). Corrosion resistance and mechanism of high-entropy alloys: A review. Mater. Corros..

[100] Sasi A., Vikram R.J., Dash K. (2025). Corrosion and oxidation behavior of high entropy alloys in extreme and harsh environments: A perspective on steam corrosion. J. Appl. Phys..

[101] Pan Y., Sun B., Wang L., Liu Z., Jiang B., Yang W., Deng Y., Li X. (2025). Investigating the correlation of microstructure with stress corrosion cracking of the high-temperature heat treated 2205 duplex stainless steel. J. Mater. Res. Technol..

[102] Wang P., Wu H., Liu X., Xu C. (2024). Machine Learning-Assisted Prediction of Stress Corrosion Crack Growth Rate in Stainless Steel. Crystals.

[103] Seghier M.E.A.B., Mohamed O.A., Ouaer H. (2024). Machine learning-based Shapley additive explanations approach for corroded pipeline failure mode identification. Structures.

[104] Hussain M., Zhang T., Chaudhry M., Jamil I., Kausar S., Hussain I. (2024). Review of Prediction of Stress Corrosion Cracking in Gas Pipelines Using Machine Learning. Machines.

[105] Zhao Z., Akhtar M.N., Bakar E.A., Abdul Razak N.B. (2025). A review on corrosion modelling for submarine pipeline. Ain Shams Eng. J..

[106] Xiong X., Zhang N., Yang J., Chen T., Niu T. (2024). Machine Learning-Assisted Prediction of Corrosion Behavior of 7XXX Aluminum Alloys. Metals.

[107] Schoell R., Xi L., Zhao Y., Wu X., Yu Z., Kenesei P., Almer J., Shayer Z., Kaoumi D. (2020). In situ synchrotron X-ray tomography of 304 stainless steels undergoing chlorine-induced stress corrosion cracking. Corros. Sci..

[108] Martínez-Pañeda E. (2024). Phase-field simulations opening new horizons in corrosion research. MRS Bull..

[109] Elkhodbia M., Barsoum I. (2025). Coupled Chemo-Thermo-Mechanical Phase Field Modeling of Hydrogen Assisted Cracking. Int. J. Hydrogen Energy.

[110] Makuch M., Kovacevic S., Wenman M.R., Martínez-Pañeda E. (2024). A microstructure-sensitive electro-chemo-mechanical phase-field model of pitting and stress corrosion cracking. arXiv.

[111] Reddy K.S., Rajagopal A., Rabczuk T. (2025). A phase-field model for stress-assisted hydrogen diffusion and fracture: An Abaqus implementation. Theor. Appl. Fract. Mech..

